# Family health coverage and care reorganization during the COVID-19 pandemic*

**DOI:** 10.1590/1518-8345.7726.4789

**Published:** 2026-02-02

**Authors:** Rodrigo das Neves Cano, Ana Paula de Vechi Corrêa, Silvia Carla da Silva André Uehara

**Affiliations:** 1 Universidade Federal de São Carlos, São Carlos, SP, Brazil

**Keywords:** COVID-19, Pandemics, Primary Health Care, Public Health Surveillance, Health Management, National Health Strategies

## Abstract

to evaluate the reorganization of Primary Health Care services for individuals with suspected and/or confirmed COVID-19 during the critical phase of the pandemic, considering family health team coverage.

analytical cross-sectional study conducted with 1,474 managers of Primary Health Care services. Data were collected using Google Forms and analyzed by prevalence ratios, employing a Poisson regression model with random effects.

municipalities with coverage below 25% showed a 10% higher prevalence of patient distancing, a 33% higher prevalence of attending patients with suspected/confirmed COVID-19 in a separate sector, a 60% higher prevalence of using Telehealth for monitoring mild cases, and a 7% higher prevalence of providing guidance on home isolation, compared to municipalities with coverage between 25% and 49.99%

the reorganization of Primary Health Care occurred differently among Family Health Strategy, with municipalities with lower coverage more frequently implementing prevention and monitoring measures. This finding underscores the need to establish and standardize protocols to guide the reorganization of health services in public health emergencies.

## Highlights

**(1)** Family health coverage influenced care during the pandemic. **(2)** Lower levels of family health coverage showed better results. **(3)** Adoption of preventive measures was more prevalent in municipalities with coverage below 25%. **(4)** Case monitoring through Telehealth was more prevalent in municipalities with coverage below 25%. **(5)** Active and continuous surveillance was less prevalent in municipalities with coverage below 25%.

## Introduction

The COVID-19 pandemic gave rise to economic, political, and social changes in several countries^(1)^. These changes contributed — and continue to contribute — to exposing the weaknesses of health systems worldwide; it should be noted that, even in the pre-pandemic period, health systems were already facing difficulties^(2)^.

Globally, Primary Health Care (PHC) played an essential role in the reorganization of health systems to confront the public health emergency caused by COVID-19. The role of PHC as the entry point into the health system, in addition to decentralizing care, engaging in health surveillance, and using information and communication technologies, were key factors for effective action during the pandemic. However, the reorganization of this level of care occurred in different ways and to varying degrees across localities^(3)^.

Health services underwent changes not only in their physical infrastructure but also in work processes and management from the onset of the pandemic, particularly within PHC. The concern with ensuring safe environments for people to move through, maintaining follow-up for patients with chronic conditions, and continuing programs in priority areas led PHC services to rethink their work approaches^(4-5)^.

The pandemic imposed on PHC, worldwide, the need to adapt services to incorporate specific surveillance and monitoring actions for COVID-19, with intensified home-based treatment, including Telemedicine and Telemonitoring, which were widely used in countries such as Israel, Spain, and Brazil^(6-8)^. In many localities, Telemonitoring within PHC was strategically important, both for monitoring patients during isolation and for encouraging care among those at higher risk of infection^(9-10)^.

As the main strategy of the health system to strengthen PHC, it is important to highlight that in Brazil the Family Health Strategy (FHS) played a prominent role in controlling user flow in health units, conducting diagnostic testing for COVID-19, distributing medications, documents, and guidance both in facilities and households, as well as implementing measures to encourage isolation and physical distancing. Within the FHS, Telemonitoring and health education initiatives proved to be important practices from the outset of the pandemic, particularly given the characteristics of this service, such as proximity and strong ties with the assigned population. This approach enabled more precise monitoring of cases^(6,11-12)^.

It has become increasingly imperative for managers to address the needs of PHC by providing basic infrastructure for service delivery and ensuring a well-trained workforce, particularly during health emergencies. Understanding the dynamics of the changes imposed by the pandemic is a key factor in reorganizing PHC in Brazil. It is also worth noting the lack of comparative studies on the reorganization of PHC according to different levels of FHS coverage during the critical phase of the pandemic.

In this context, the literature — both in Brazil and internationally — demonstrates how PHC reorganized itself during the pandemic^(6,11-16)^. However, most of these studies were conducted during the first year of the pandemic, offering more of a snapshot; in contrast, this study resembles a film, that is, a longitudinal analysis of the critical phase of the pandemic. Thus, this study presents pioneering results, gaining relevance in the post-pandemic context by directly addressing the knowledge gap on how PHC services were reorganized (structurally, in care delivery, and in surveillance) from the perspective of service managers, in a comparative analysis that considers different levels of FHS coverage. In doing so, it provides scientific evidence to inform decision-making and health planning, particularly in preparation for future public health emergencies.

Accordingly, the objective of this study was to evaluate the reorganization of Primary Health Care for individuals with suspected and/or confirmed COVID-19 during the critical phase of the pandemic, considering FHS coverage.

## Method

### Study design

This is an analytical cross-sectional study, written in accordance with the Strengthening the Reporting of Observational Studies in Epidemiology (STROBE) guidelines.

### Population and sample

The target population of this study consisted of managers of PHC services in Brazil. It is important to note that, due to the absence of official data on the number of managers in these services, convenience sampling was adopted. In this context, the initial database included 1,651 participants. From this total, duplicate answers (n=30), incomplete answers (n=14), and forms completed by professionals who were not PHC service managers (n=133) were excluded, resulting in a final sample of 1,474 PHC service managers from Brazilian municipalities.

Inclusion criteria were professionals responsible for PHC services for at least three months during the public health emergency imposed by the COVID-19 pandemic. Incomplete forms were excluded.

### Data collection

Data were collected remotely, due to the COVID-19 pandemic period and the large number of participants, between April and September 2022, through a self-administered questionnaire in Google Forms. The extended data collection period was justified by the difficulty in reaching participants from all regions.

The questionnaire was developed by the researchers and subsequently reviewed by three field experts to assess its relevance and adequacy; however, no psychometric evaluation was performed. A pilot test was conducted in a municipality in the state of São Paulo, during which the researchers analyzed the presence of difficult, ambiguous, or poorly formulated questions. This process allowed for improvements to enhance comprehension. The pilot test was applied to 49 participants, and its results were included in the final sample.

The instrument was developed based on the Clinical Management Protocol for Coronavirus (COVID-19) in PHC from the Brazilian Ministry of Health^(17)^, and included the following variables: measures to prevent contagion in health units; stratification of influenza-like syndrome severity; therapeutic management; referral to urgent/emergency or hospital services for severe cases; clinical monitoring; community prevention measures; and support for active surveillance.

Data collection was conducted by a team of five collectors, who initially distributed the questionnaire link to email addresses publicly available on the official websites of municipal health departments. To increase adherence, the study was supported by the National Council of Health Secretaries (CONASS) and the National Council of Municipal Health Secretaries (CONASEMS), which disseminated and emphasized the importance of participation in the survey among municipal health departments. Additionally, staff from the Municipal Health Secretaries’ Councils collaborated in promoting the study through the Regional Health Departments. These measures were adopted to reach the largest possible number of professionals from all Brazilian regions, ensuring equal opportunity for participation.

### Data analysis

For the analysis of FHS coverage, no ideal parameter for service coverage was identified in the literature. Accordingly, the researchers defined the following classification: <25%, 25.01%–49.99%, 50%–74.99%, and >75%. The FHS coverage of the participating municipalities was obtained from the Ministry of Health’s *e-Gestor AB* platform^(18)^ and considered the exposure variable, while the outcome variables referred to the reorganization of PHC services, classified as composite variables.

Initially, data were described using absolute and relative frequencies (qualitative variables) and measures such as mean, standard deviation, minimum, median, and maximum (quantitative variables).

To estimate prevalence ratios (PR) and confidence intervals (95% CI) when comparing FHS coverage ranges, a Poisson regression model with random effects was applied^(19)^. All analyses were conducted using SAS software, version 9.4. A 5% significance level was adopted for all tests.

### Ethical aspects

The study was approved by the Ethics Committee of the Federal University of São Carlos, CAAE 52527521.8.0000.5504.

## Results

A total of 1,474 PHC service managers from municipalities across Brazil participated in the study. Of these, 45.9% (n=676) were from the Southeast region, 21% (n=311) from the Northeast, 17.5% (n=258) from the South, 11.7% (n=173) from the Midwest, and 3.8% (n=56) from the North. In terms of gender, 86.6% (n=1,276) identified as female and 13.4% (n=198) as male, with a mean age of 38.9 years. Although the study included a high proportion of participants from the Southeast region, which may have introduced prevalence bias, the sample encompassed participants from all states, providing consistent and robust results regarding the reorganization of Brazilian PHC during the critical phase of the pandemic.

Across comparisons by FHS coverage, the adoption of preventive measures against contagion in health units was reported by all participants. Among these measures, patient-to-patient distancing was 10% more prevalent (CI: 1.03; 1.17) in municipalities with FHS coverage below 25% compared to those with coverage between 25% and 49.99%; 8% higher (CI: 1.03; 1.13) compared to municipalities with coverage between 50% and 74.99%; and 9% higher (CI: 1.05; 1.14) compared to municipalities with coverage of 75% or more. Cleaning and disinfecting equipment between patients was 12% more prevalent (CI: 1.02; 1.22) in municipalities with coverage below 25% compared to those with coverage between 50% and 74.99% (Table 1).

All participants reported that health services had the capacity to attend patients with suspected or confirmed COVID-19 in a separate ward, with prevalence being 33% higher (CI: 1.04; 1.70) in municipalities with FHS coverage below 25% compared to those with coverage between 25% and 49.99% (Table 1).

**Table 1-:** Comparisons among municipalities, according to FHS* coverage, regarding the adoption of preventive measures against contagion in health units. São Carlos, SP, Brazil, 2022

**Variable**	**Comparisons**					
	**<25% vs 25%–49.99%**		**<25% vs 50%–74.99%**		**<25% vs >75%**	
	**PR** ^†^ **(95% CI)** ^‡^	**p-value** ^§^	**PR** ^†^ **(95% CI)** ^‡^	**p-value** ^§^	**PR** ^†^ **(95% CI)** ^‡^	**p-value** ^§^
Given the possibility that the patient (and companion) may be infected with COVID-19, are infection prevention measures implemented in the Health Unit?						
No	-	-	-	-	-	-
Yes	1 (1; 1)	-	1.01 (1; 1.01)	0.12	1 (1; 1.01)	0.16
If yes, please indicate which: ^||^						
Identification of symptoms (such as cough, runny nose, and difficulty breathing)	0.98 (0.92; 1.05)	0.60	0.97 (0.92; 1.03)	0.37	0.99 (0.93; 1.05)	0.68
Guidance on and availability of surgical mask use	1.04 (0.94; 1.14)	0.45	1.03 (0.96; 1.11)	0.37	1.05 (0.98; 1.13)	0.14
Distancing between one patient and another	1.1 (1.03; 1.17)	<0.01	1.08 (1.03; 1.13)	<0.01	1.09 (1.05; 1.14)	<0.01
Separate wards for patients who reported symptoms similar to COVID-19	1.12 (0.81; 1.54)	0.50	0.83 (0.62; 1.12)	0.23	1.08 (0.85; 1.37)	0.52
Cleaning and disinfection of equipment used from one patient to another	1.07 (0.99; 1.16)	0.10	1.12 (1.02; 1.22)	0.01	1.05 (0.99; 1.12)	0.12
Are there conditions to treat patients with suspected or confirmed COVID-19 in a ward separate from other patients?						
No	-	-	-	-	-	-
Yes	1.33 (1.04; 1.7)	0.02	0.97 (0.75; 1.26)	0.82	1.08 (0.89; 1.31)	0.44

*FHS = Family Health Strategy; ^†^PR = Prevalence Ratio; ^‡^95% CI = 95% Confidence Interval; ^§^p-value = Significance level; ^||^More than one answer per participant was allowed

With respect to preventive measures against contagion adopted in health units, comparisons among municipalities with FHS coverage between 25% and 49.99%, 50% and 74.99%, and greater than 75% showed that the availability of separate wards for patients reporting symptoms similar to COVID-19 was 30% more prevalent (CI: 1.05; 1.61) in municipalities with 50%–74.99% coverage compared to those with coverage above 75% (Table 2).

Regarding the conditions for treating patients with suspected or confirmed COVID-19 in a ward separate from other patients, prevalence was 27% lower (CI: 0.57; 0.94) in municipalities with 25%–49.99% FHS coverage compared to those with 50%–74.99% coverage. In the comparison between municipalities with 25%–49.99% coverage and those with more than 75% coverage, this same measure was 19% less prevalent (0.68; 0.97) in municipalities with lower coverage (Table 2).

**Table 2-:** Comparisons among municipalities, according to FHS* coverage, regarding the adoption of preventive contagion measures in health units. São Carlos, SP, Brazil, 2022

**Variable**	**Comparisons**					
	**25%** – **49.99% vs. 50%** – **74.99%**		**25%** – **49.99% vs. >75%**		**50%** – **74.99% vs. >75%**	
	**PR** ^†^ **(95% CI)** ^‡^	**p-value** ^§^	**PR** ^†^ **(95% Ci)** ^‡^	**p-value** ^§^	**PR** ^†^ **(95% CI)** ^‡^	**p-value** ^§^
Given the possibility that the patient (and companion) may be infected with COVID-19, are infection prevention measures implemented in the Health Unit?						
No	-	-	-	-	-	-
Yes	1.01 (1; 1.01)	0.12	1 (1; 1.01)	0.16	1 (0.99; 1)	0.38
If yes, please indicate which: ^||^						
Identification of symptoms (such as cough, runny nose, and difficulty breathing)	0.99 (0.96; 1.03)	0.57	1 (0.97; 1.04)	0.78	1.02 (0.99; 1.04)	0.24
Guidance on and availability of surgical mask use	1 (0.92; 1.08)	0.90	1.02 (0.94; 1.1)	0.70	1.02 (0.98; 1.06)	0.32
Distancing between one patient and another	0.98 (0.92; 1.05)	0.63	1 (0.94; 1.06)	0.91	1.01 (0.97; 1.06)	0.57
Separate wards for patients who reported symptoms similar to COVID-19.	0.75 (0.55; 1.01)	0.06	0.97 (0.76; 1.24)	0.79	1.3 (1.05; 1.61)	0.02
Cleaning and disinfection of equipment used from one patient to another	1.04 (0.95; 1.14)	0.37	0.98 (0.92; 1.05)	0.56	0.94 (0.88; 1.01)	0.09
Are there conditions to treat patients with suspected or confirmed COVID-19 in a ward separate from other patients?						
No	-	-	-	-	-	-
Yes	0.73 (0.57; 0.94)	0.01	0.81 (0.68; 0.97)	0.02	1.11 (0.91; 1.35)	0.29

*FHS = Family Health Strategy; ^†^PR = Prevalence Ratio; ^‡^95% CI = 95% Confidence Interval; ^§^p-value = Significance level; ^||^More than one answer per participant was allowed

Regarding the measures adopted in the management of mild COVID-19 cases, hydration guidance was 8% more prevalent (CI: 1.03; 1.14) in municipalities with FHS coverage below 25% compared to those with 25%–49.99% coverage, and 6% more prevalent (CI: 1.01; 1.10 and CI: 1.03; 1.10) compared to municipalities with coverage above 50%. Nutritional guidance was 14% more prevalent (CI: 1.06; 1.24) in municipalities with coverage below 25% compared to those with coverage between 25% and 49.99%, and 9% more prevalent (CI: 1.01; 1.16) municipalities with coverage below 25% compared to those with coverage above 75% (Table 3).

Guidance on home isolation was 7% (CI: 1.02; 1.12) more prevalent in municipalities with FHS coverage below 25% compared to those with coverage between 25% and 49.99%, and 4% (CI:1.01; 1.07) more prevalent in municipalities with coverage below 25% compared to those with coverage above 50%. The use of analgesics was 15% (CI: 1.01; 1.31) more prevalent in municipalities with coverage below 25% compared to those with coverage between 25% and 49.99%. Telehealth for monitoring mild COVID-19 cases was 60% (CI: 1.21; 2.11) more prevalent in municipalities with coverage below 25% compared to those with coverage between 25% and 49.99% (Table 3).

Active and continuous surveillance of followed patients was 21% (CI: 0.66; 0.93) less prevalent in municipalities with coverage below 25% compared to those with coverage above 75%. Symptom review and follow-up every 48 hours, preferably by phone, showed a similar result, being 21% (CI: 0.63; 0.99) less prevalent in municipalities with coverage below 25% compared to those with coverage above 75% (Table 3).

It is also noteworthy that, regarding the verification of bed availability in the reference hospital for the referral of patients requiring hospitalization, the prevalence was 32% (CI: 0.48–0.96) lower in municipalities with FHS coverage of less than 25% compared to those with coverage greater than or equal to 75% (Table 3).

**Table 3-:** Comparisons between municipalities, according to FHS* coverage, regarding measures for managing mild cases, monitoring and active continuous surveillance, and verification of bed availability before referral. São Carlos, SP, Brazil, 2022

**Variable**	**Comparisons**					
	**<25% vs 255** – **49.99%**		**<25% vs 50%** – **74.99%**		**<25% vs >75%**	
	**PR** ^†^ **(95% CI)** ^‡^	**p-value** ^§^	**PR** ^†^ **(95% CI)** ^‡^	**p-value** ^§^	**PR** ^†^ **(95% CI)** ^‡^	**p-value** ^§^
What measures were adopted for the management of mild COVID-19 cases? ^||^						
Rest guidance	1.03 (0.96; 1.1)	0.46	1 (0.95; 1.06)	0.92	1.02 (0.96; 1.07)	0.58
Hydration guidance	1.08 (1.03; 1.14)	<0.01	1.06 (1.01; 1.1)	0.01	1.06 (1.03; 1.1)	<0.01
Nutrition guidance	1.14 (1.06; 1.24)	<0.01	1.06 (0.99; 1.15)	0.11	1.09 (1.01; 1.16)	0.02
Home isolation	1.07 (1.02; 1.12)	<0.01	1.04 (1.01; 1.07)	0.02	1.04 (1.01; 1.07)	0.01
Analgesics	1.15 (1.01; 1.31)	0.04	1 (0.9; 1.12)	0.94	1.08 (0.99; 1.17)	0.07
Antipyretics	1.16 (1; 1.35)	0.05	1.03 (0.91; 1.17)	0.63	1.07 (0.98; 1.18)	0.13
Home isolation for 14 days from the date of symptom onset	0.94 (0.78; 1.13)	0.49	1.01 (0.81; 1.24)	0.96	1.02 (0.87; 1.21)	0.79
Did the health unit provide Telehealth for monitoring mild COVID-19 cases?						
No	-	-	-	-	-	-
Yes	1.6 (1.21; 2.11)	<0.01	1.17 (0.94; 1.45)	0.16	1.15 (0.99; 1.34)	0.08
Does the health unit carry out active and continuous surveillance of patients under follow-up?						
No	-	-	-	-	-	-
Yes	1.03 (0.8; 1.32)	0.81	0.86 (0.7; 1.07)	0.17	0.79 (0.66; 0.93)	<0.01
Does the health unit review symptoms and monitor disease progression every 48 hours, preferably by telephone, requesting an in-person consultation when a physical examination is necessary?						
No	-	-	-	-	-	-
Yes	1.16 (0.85; 1.58)	0.35	0.96 (0.69; 1.33)	0.79	0.79 (0.63; 0.99)	0.04
Does the health unit check whether the referral hospital for COVID-19 hospitalizations has sufficient and available beds to receive the patient requiring hospitalization before referring them?						
No	-	-	-	-	-	-
Yes	1.23 (0.74; 2.04)	0.42	0.83 (0.56; 1.23)	0.35	0.68 (0.48; 0.96)	0.03

*FHS = Family Health Strategy; ^†^PR = Prevalence Ratio; ^‡^95% CI = 95% Confidence Interval; ^§^p-value = Significance level; ^||^More than one answer per participant was allowed

Regarding the measures adopted in the management of mild COVID-19 cases, nutrition guidance was 7% (CI: 0.87; 0.99) less prevalent in municipalities with FHS coverage between 25% and 49.99% than in those with coverage between 50% and 74.99%. Telehealth for monitoring mild COVID-19 cases was 27% less prevalent (CI: 0.55; 0.98) in municipalities with coverage between 25% and 49.99% compared to those with coverage between 50% and 74.99%; and 28% less prevalent (CI: 0.56; 0.92) in municipalities with coverage between 25% and 49.99% compared to those with coverage above 75% (Table 4).

Active and continuous surveillance of followed patients was 24% less prevalent (CI: 0.62;0.94) in municipalities with FHS coverage between 25% and 49.99% than in those with coverage greater than 75%; Similarly, the review of symptoms and monitoring of disease progression every 48 hours, preferably by telephone, was 32% lower (CI: 0.54;0.87) in municipalities with coverage between 25% and 49.99% than in those with coverage greater than 75% (Table 4).

Finally, checking the availability of beds in referral hospitals before transferring patients requiring hospitalization was 45% less prevalent (CI: 0.38;0.81) in municipalities with coverage between 25% and 49.99% compared to those with coverage above 75% (Table 4).

**Table 4-:** Comparisons between municipalities, according to FHS* coverage, regarding measures for managing mild cases, monitoring and active continuous surveillance, and verification of bed availability before referral. São Carlos, SP, Brazil, 2022

**Variable**	**Comparisons**					
	**25%** – **49.99% vs. 50%** – **74.99%**		**25%** – **49.99% vs. >75%**		**50%** – **74.99% vs. >75%**	
	**PR** ^†^ **(95% CI)** ^‡^	**p-value** ^§^	**PR** ^†^ **(95% CI)** ^‡^	**p-value** ^§^	**PR** ^†^ **(95% CI)** ^‡^	**p-value** ^§^
What measures were adopted for the management of mild COVID-19 cases? ^||^						
Rest guidance	0.98 (0.92; 1.04)	0.44	0.99 (0.94; 1.04)	0.68	1.01 (0.97; 1.05)	0.54
Hydration guidance	0.97 (0.93; 1.02)	0.25	0.98 (0.94; 1.02)	0.32	1.01 (0.97; 1.04)	0.72
Nutrition guidance	0.93 (0.87; 0.99)	0.03	0.95 (0.9; 1.01)	0.08	1.02 (0.97; 1.08)	0.41
Home isolation	0.97 (0.92; 1.01)	0.16	0.97 (0.92; 1.01)	0.17	1 (0.98; 1.03)	0.93
Analgesics	0.88 (0.76; 1.01)	0.06	0.94 (0.84; 1.06)	0.32	1.08 (0.98; 1.18)	0.12
Antipyretics	0.89 (0.76; 1.03)	0.12	0.92 (0.81; 1.05)	0.22	1.04 (0.94; 1.15)	0.41
Home isolation for 14 days from the date of symptom onset	1.07 (0.91; 1.26)	0.40	1.09 (0.99; 1.2)	0.08	1.02 (0.88; 1.17)	0.81
Did the health unit provide Telehealth for monitoring mild COVID-19 cases?						
No	-	-	-	-	-	-
Yes	0.73 (0.55; 0.98)	0.04	0.72 (0.56; 0.92)	<0.01	0.98 (0.82; 1.18)	0.85
Does the health unit carry out active and continuous surveillance of patients under follow-up?						
No	-	-	-	-	-	-
Yes	0.83 (0.65; 1.07)	0.15	0.76 (0.62; 0.94)	<0.01	0.91 (0.78; 1.07)	0.26
Does the health unit review symptoms and monitor disease progression every 48 hours, preferably by telephone, requesting an in-person consultation when a physical examination is necessary?						
No	-	-	-	-	-	-
Yes	0.82 (0.58; 1.16)	0.27	0.68 (0.54; 0.87)	<0.01	0.83 (0.63; 1.08)	0.17
Does the health unit check whether the referral hospital for COVID-19 hospitalizations has sufficient and available beds to receive the patient requiring hospitalization before referring them?						
No	-	-	-	-	-	-
Yes	0.68 (0.44; 1.03)	0.07	0.55 (0.38; 0.81)	<0.01	0.82 (0.67; 1)	0.05

*FHS = Family Health Strategy; ^†^PR = Prevalence Ratio; ^‡^95% CI = 95% Confidence Interval; ^§^p-value = Significance level; ^||^More than one answer per participant was allowed

## Discussion

The results of this study demonstrate that FHS coverage influenced the reorganization of work processes during the critical phase of the COVID-19 pandemic. Municipalities with FHS coverage below 25% showed higher prevalence rates in the implementation of COVID-19 prevention and control measures, as well as in the reorganization of clinical management of mild cases and work processes.

In this context, more populous municipalities tend to have lower FHS coverage compared to smaller ones. This reduced coverage in large urban centers is associated with greater complexity and higher population density, which hinder the implementation and universalization of PHC. However, this does not imply a lack of effort, as many of these municipalities compensate with a broader variety of services and more complex and robust networks^(20)^.

Conversely, smaller municipalities tend to have higher FHS coverage, as do vulnerable areas within medium- and large-sized municipalities^(20)^, which were the most affected by the spread of the disease during the critical phase of the pandemic. At that time, faster and more effective actions from public management were required to reorganize PHC flows and to refer cases of clinical worsening due to COVID-19.

The findings of this study suggest that larger municipalities, with lower FHS coverage, reorganized PHC services in a more practical manner to optimize the available resources, which are generally characterized by low technological density and shortages of human resources. In this scenario, municipalities in São Paulo with more than 50,000 inhabitants instructed all PHC services to attend patients with COVID-19, whether or not they had exclusive units dedicated to managing the disease^(21)^, which inherently required professionals to adapt to the reality of that moment. Similarly, cities in Latin America, particularly in Chile and Colombia, reorganized care for COVID-19 patients within dedicated units while also leveraging the existing PHC structure^(22)^.

Among the measures adopted to reorganize the PHC, noteworthy actions included prevention initiatives, the allocation of separate wards for symptomatic patients, and the reorganization of internal flows, all of which contributed to controlling COVID-19. In the initial phase, measures such as isolation, quarantine, physical distancing, and flow control proved effective in reducing transmission and flattening the infection curve^(23)^. Countries such as Italy, France, Germany, and Spain, which implemented strict lockdowns and widespread testing, were able to reduce lethality and contain viral mutations. In contrast, Sweden, where lockdown measures were less stringent, saw the emergence of new viral mutations^(24)^.

Since COVID-19 is transmitted via respiratory pathways, particularly in crowded settings, reorganizing patient flow within health services was essential. Separating users with suspected or confirmed infection from others reduced the risk of transmission. This reorganization also enabled safer case management, protecting both health professionals and patients while ensuring the continuity of services in a safe and efficient manner^(25)^. In Canada, the rapid establishment of isolation centers within health units yielded positive results early in the pandemic^(26)^. In France, PHC services created specific waiting areas and corridors for symptomatic patients^(16)^.

It is also noteworthy that municipalities with lower FHS coverage, larger population size, and higher density may have centralized care for individuals with symptoms and/or COVID-19 in specific services, reorganizing FHS to ensure continuity of care and preserve integrality, longitudinality, and access during the health emergency. Moreover, these municipalities had greater capacity to organize services across all levels, with more resources to mobilize equipment, supplies, personnel, and alternative arrangements in the functioning of health services^(27)^.

The COVID-19 emergency demanded swift action from health systems worldwide, which initially focused on ensuring hospital bed availability for the population^(28)^. However, alongside or shortly after the reorganization of hospital care, an immediate restructuring of PHC services became necessary, as hospitals became overwhelmed—as observed in the early collapse in Italy^(29)^, and it was found that most cases were of low complexity, enabling care to be decentralized through PHC services, which until then had remained underutilized^(30)^.

It is worth noting that PHC must have high resoluteness capacity in meeting basic health needs and be effective in reducing hospitalizations for conditions sensitive to primary care. However, its underutilization during the pandemic worsened the preexisting crisis in a historically underfunded system, contributing to resource shortages. The weakness of this foundation compromised the monitoring of COVID-19 cases and the implementation of effective active surveillance measures, leading to increased hospitalizations and overburdening emergency services. Strengthening PHC could have generated a more efficient response and mitigated the impact of the pandemic in Brazil^(28)^.

The attributes of PHC, including the FHS — such as territorial responsibility, user linkage, and continuity of care — were underutilized in many Brazilian municipalities, particularly during the first year of the pandemic. During this period, there was a significant reduction in the use of preventive and therapeutic health services, attributed to mobility restrictions, physical distancing, and fear of infection^(31)^.

Despite the delayed redirection of PHC toward addressing the pandemic, services gradually adapted and strengthened until they assumed a central role in care during the health emergency. The adaptability and innovative capacity of PHC made it possible to efficiently reorganize user flow and manage new clinical protocols, reinforcing the importance of strengthening PHC to ensure continuity of care and the management of health conditions beyond the emergency^(32)^.

During the critical phase of the pandemic, the adaptation of PHC and the intensification of work revealed marked differences among municipalities due to the absence of national guidelines. Local and regional inequalities in PHC infrastructure — such as human resources, access to supplies, technologies, and coordination capacity — exacerbated unequal responses to the crisis. Municipalities with greater FHS coverage, structured management, and integration with epidemiological surveillance demonstrated better responses, whereas localities with low technical density or historical weaknesses faced greater challenges in organizing care and managing COVID-19 cases. These differences were further intensified by the lack of unified protocols, which forced managers and professionals to make local decisions in the midst of uncertainty^(3,33)^.

Beyond clinical management, pain management and guidance on adequate nutrition and hydration cannot be dissociated from health education, which constitutes one of the fundamental pillars of the FHS. During the pandemic, health education was essential not only to promote disease prevention measures — such as mask use and physical distancing — but also to combat misinformation about the virus and vaccines, provide guidance on symptom identification and home care, reduce the stigma associated with infection, and offer support for mental health^(34)^.

It is important to note that the FHS provides care directed toward priority groups defined by public health policies, including children, older adults, women, individuals with hypertension, diabetes, and obesity, as well as those with mental health needs^(35)^. In this context, COVID-19 — considered an emerging disease and currently endemic — must be incorporated into the scope of FHS activities. This means that management should seek ways to train health professionals to address this new demand, so they can deliver comprehensive and effective care, in addition to surveillance actions, regardless of municipal population coverage.

Furthermore, the results of this study showed that the surveillance of suspected or confirmed cases was more frequent in municipalities with greater FHS coverage. One explanation for this success may lie in the composition of the FHS teams, particularly the work of Community Health Agents (CHAs). The work of CHAs was decisive for the success of community surveillance during the pandemic, as they promoted territorialized and continuous care — even in adverse and high-demand scenarios — expanding the reach of surveillance actions, particularly in vulnerable areas with limited access to health services^(36-37)^.

The effectiveness of FHS teams in the active surveillance of COVID-19 cases, and even in the direct provision of care to these patients, was closely linked to the use of information and communication technologies, such as Telemedicine and digital platforms. These tools facilitated virtual consultations, remote monitoring, and the dissemination of information, thereby enhancing both accessibility and efficiency of care. These integrated actions were essential to controlling the spread of the virus and ensuring continuity of care^(38)^. Municipalities with greater FHS coverage are, for the most part, small- and medium-sized, where health services are often the only ones available in the municipality. These municipalities demonstrate stronger control and effectiveness in surveillance actions due to their access to many households, close ties with the population, and demand for services. Thus, this characteristic may explain the greater adherence of these health services to surveillance actions, follow-up on the progression of users’ clinical conditions, and the use of Telemedicine and Telemonitoring.

The effective health surveillance actions, implemented more frequently in municipalities with broad FHS coverage, not only supported the monitoring and control of COVID-19 but also contributed to the proper referral of severe cases, optimizing the use of available hospital beds. In this context, the importance of coordination across different points of the Health Care Network (RAS, acronym for *Rede de Atenção à Saúde*) should be emphasized, particularly the integration between PHC and medium- and high-complexity services. The continuous flow of information, combined with the training and qualification of health professionals, proved essential for the efficient use of existing resources, especially in times of scarcity^(39)^. In another context, in Norway, integration between PHC services and other health services was observed through unified protocols and guidelines, ensuring safe care for all patients^(40)^.

However, the pandemic exacerbated existing inequalities in the distribution of hospital beds among Brazilian municipalities, as regions with lower socioeconomic conditions faced greater difficulties in accessing hospitalization, resulting in higher rates of delayed admissions and mortality. The centralization of hospital resources in state capitals and medium-sized cities hindered timely access to intensive care for populations living in remote or peripheral areas. This situation exposed long-standing structural weaknesses of the Unified Health System (SUS, acronym in Portuguese) and highlighted the urgency of a more equitable redistribution of health resources, particularly regarding hospital infrastructure^(41)^.

Given this scenario, it becomes essential to strengthen PHC, not only as the system’s entry point but also as the coordinator of care, with expanded problem-solving capacity and effective clinical management strategies. The qualified performance of FHS teams, aligned with well-defined clinical protocols, can contribute to accurate case triage, ensure that referrals to the hospital level occur in a timely and rational manner, based on severity, and help reduce hospital overload and adverse outcomes^(42-43)^.

Finally, the results of this study show that the presence of FHS teams enabled a rapid and adaptive response, facilitating the management of patients with COVID-19, the promotion of preventive measures, and the use of communication technologies such as Telemedicine. It is noteworthy that these technologies remained significantly in place after the pandemic, especially in contexts with greater digital maturity, training in digital health, and favorable infrastructure, representing an important legacy not only in Brazil but also worldwide^(44)^.

As in Brazil, the pandemic accelerated transformations in PHC in Canada, driving the expansion of digital initiatives such as online scheduling, as well as emergency services like COVID-19 specialty clinics and Telehealth. These changes left a legacy regarding the consolidation of Telehealth and the need for more agile and integrated models capable of responding to crises without compromising access to care^(45)^. Both cases reinforce the strategic role of PHC and digital infrastructure in public health emergencies.

This study presents limitations inherent to its cross-sectional design, such as the absence of causality and the potential for prevalence bias. The predominance of municipalities from the Southeast region in the sample may have introduced a prevalence bias. In addition, the use of self-administered questionnaires for data collection may have resulted in response bias, due to possible misinterpretation of questions and over- or underestimation of the information provided by participants. Nevertheless, the sample includes representatives from all states and provides consistent and robust results regarding the reorganization of Brazilian PHC during the critical phase of the pandemic, particularly in relation to FHS coverage.

## Conclusion

The FHS played an essential role during the critical phase of the COVID-19 pandemic by ensuring continuous and effective support for the population. The development of preventive actions, monitoring of mild cases, and community guidance contributed to reducing the spread of the virus and managing the population’s health needs. In addition, the FHS helped maintain continuity of care for chronic conditions and other health demands, underscoring its importance in responding to public health emergencies in an adaptable and sustainable manner.

Future studies are recommended to assess the long-term impact of the reorganization of PHC during the pandemic on team work processes. Furthermore, the findings highlight the importance of creating and standardizing protocols to guide the reorganization of health services in public health emergencies.
