# Supplementary material for: Family health coverage and care reorganization during the COVID-19 pandemic
Source: Rev Lat Am Enfermagem. 2026 Feb 2;34:e4789. doi: 10.1590/1518-8345.7726.4789 (PMC12863657; doi:10.1590/1518-8345.7726.4789)
Supplement: Translation

# Translation: Cobertura de saúde da família e a reorganização da assistência durante a pandemia de COVID-19*

**DOI:** 10.1590/1518-8345.7726.4790

**Authors:** Rodrigo das Neves Cano, Ana Paula de Vechi Corrêa, Silvia Carla da Silva André Uehara

**Affiliations:** 1 Universidade Federal de São Carlos, São Carlos, SP, Brasil

**Keywords:** COVID-19, Pandemias, Atenção Primária à Saúde, Vigilância em Saúde Pública, Gestão em Saúde, Estratégias de Saúde Nacionais

## Abstract

avaliar a reorganização da assistência da Atenção Primária à Saúde às pessoas com suspeita e/ou diagnóstico de COVID-19 durante a fase crítica da pandemia, considerando a cobertura de equipe de saúde da família.

estudo transversal analítico, realizado com 1.474 gerentes de serviços da Atenção Primária. Os dados foram coletados no *Google Forms* e analisados por razões de prevalência, utilizando modelo de regressão de Poisson com efeito aleatório.

municípios com cobertura menor que 25% apresentaram prevalência 10% maior no distanciamento de pacientes, 33% maior no atendimento de pacientes com suspeita/diagnóstico de COVID-19 em setor separado, 60% maior no uso de teleatendimento para monitoramento dos casos leves e 7% maior orientação sobre isolamento domiciliar, quando comparados aos municípios com cobertura entre 25% e 49,99%.

a reorganização da assistência da Atenção Primária à Saúde aconteceu de forma distinta entre as Estratégias de Saúde da Família, sendo que municípios com menores coberturas implementaram medidas de prevenção e monitoramento com maior frequência. Este fato aponta a necessidade da criação e padronização de protocolos que orientem a reorganização dos serviços de saúde em situações de emergência sanitária.

## Destaques

**(1)** Cobertura de saúde da família influenciou a assistência durante a pandemia. **(2)** Menores coberturas de saúde da família apresentaram melhores resultados. **(3)** Adoção de medidas de prevenção foi mais prevalente em cobertura menor que 25%. **(4)** Monitoramento por teleatendimento foi mais prevalente em cobertura menor que 25%. **(5)** A vigilância ativa e continuada foi menos prevalente em cobertura menor que 25%.

## Introdução

A pandemia de COVID-19 fez emergir, em diversos países, mudanças econômicas, políticas e sociais^(1)^. Tais mudanças contribuíram e ainda cooperam para evidenciar as fragilidades dos sistemas de saúde pelo mundo; ressalta-se que, no período pré-pandêmico, os sistemas de saúde já enfrentavam dificuldades^(2)^.

Em todo o mundo, a Atenção Primária à Saúde (APS) foi essencial durante o processo de readequação dos sistemas de saúde para o enfrentamento da emergência sanitária causada pela COVID-19. A atuação da APS como porta de entrada do sistema, além da descentralização dos cuidados em saúde, envolvimento com a vigilância em saúde, utilização de tecnologias de informação e comunicação, foram os pontos-chave para uma ação efetiva na pandemia; entretanto, a reorganização desse nível de assistência foi de forma e intensidade diferente em cada localidade^(3)^.

Os serviços de saúde sofreram alterações em suas instalações físicas e também em todo o processo de trabalho e gestão, desde o início da pandemia, em especial na APS. A preocupação em garantir ambientes seguros para que as pessoas pudessem circular, manter o acompanhamento de usuários com condições crônicas, além de continuar programas nas áreas prioritárias, levou os serviços da APS a repensarem as formas de trabalho^(4-5)^.

A pandemia impôs à APS, em âmbito mundial, a necessidade de adequação do serviço para incorporar ações de vigilância e monitoramento específicas para a COVID-19, tratamento domiciliar intensificado, incluindo a telemedicina e o telemonitoramento, muito utilizado em países como Israel, Espanha e até mesmo no Brasil^(6-8)^. Em muitas localidades, o telemonitoramento na APS foi estrategicamente importante, tanto para monitorar pacientes durante o período de isolamento quanto para estimular o cuidado em pacientes com maior risco de infecção^(9-10)^.

Como principal estratégia do sistema de saúde vigente para o fortalecimento da APS, compete destacar que, no Brasil, a Estratégia de Saúde da Família (eSF) se destacou no controle de fluxo de usuários nas unidades, realização de testes diagnósticos para COVID-19, distribuição de medicamentos, documentos e orientações na unidade e nos domicílios, além de ações para estimular o isolamento e distanciamento físico. Ainda, na eSF, o telemonitoramento e ações de educação em saúde se mostraram práticas importantes desde o início da pandemia, especialmente pelas características desse serviço, como a proximidade e vínculo com a população adscrita, viabilizando um monitoramento mais preciso dos casos^(6,11-12)^.

É cada vez mais imperativo que o gestor tenha um olhar direcionado às necessidades da APS, fornecendo infraestrutura básica para a realização de atividades, bem como mantenha sua força de trabalho capacitada, especialmente durante emergências sanitárias. Compreender como foi a dinâmica de mudanças impostas pela pandemia se reflete como um aliado importante na reorganização da APS no Brasil. Ainda cabe destacar a ausência de estudos comparativos sobre a reorganização da APS segundo diferentes níveis de cobertura de eSF durante a fase crítica da pandemia.

Nesse contexto, a literatura mostra, tanto no Brasil quanto em outros países, como a APS foi se organizando durante a pandemia^(6,11-16)^; entretanto, ressalta-se que a maioria desses estudos foram realizados durante o primeiro ano da pandemia, conotando o sentido de uma fotografia; ao contrário deste estudo, que se assemelha a um filme, ou seja, uma análise longitudinal da fase crítica da pandemia. Assim, este estudo apresenta resultados de vanguarda, tendo notoriedade no pós-pandemia, pois contribui diretamente para preencher a lacuna no conhecimento de como os serviços da APS foram reorganizados, desde o aspecto estrutural, assistencial e de vigilância, sob a ótica dos gerentes dos serviços, de uma forma comparativa, considerando as coberturas de eSF, fornecendo evidências científicas que subsidiem a tomada de decisões e o planejamento em saúde, especialmente para novas situações de emergência sanitária.

Sendo assim, este estudo teve como objetivo avaliar a reorganização da assistência da Atenção Primária à Saúde às pessoas com suspeita e/ou diagnóstico de COVID-19 durante a fase crítica da pandemia, considerando a cobertura de eSF.

## Método

### Delineamento do estudo

Trata-se de um estudo de corte transversal analítico, redigido de acordo com a ferramenta *Strengthening the Reporting of Observational Studies in Epidemiology* (STROBE).

### População e amostra

A população-alvo deste estudo foi composta por gerentes de serviços da APS do Brasil. Cabe destacar que, devido à ausência de dados oficiais sobre o número de gerentes nesses serviços, tornou-se necessária a utilização da amostragem por conveniência. Nesse contexto, inicialmente o banco de dados contou com 1651 participantes, sendo excluídas as respostas duplicadas (n= 30), respostas incompletas (n= 14) e formulários respondidos por profissionais que não eram gerentes de serviços da APS (n= 133), compondo uma amostra final de 1474 gerentes de serviços de saúde da APS de municípios brasileiros.

Adotaram-se como critério de inclusão profissionais responsáveis por serviços da APS, por pelo menos 3 meses, durante a fase de emergência sanitária imposta pela pandemia de COVID-19; e foram excluídos formulários incompletos.

### Coleta de dados

Os dados foram coletados remotamente, devido ao período da pandemia de COVID-19 e pelo elevado número de participantes, no período de abril a setembro de 2022, por meio de um questionário autorrespondido no *Google Forms*. O longo período da coleta de dados se justifica pela dificuldade de alcançar participantes de todas as regiões.

O questionário foi construído pelos próprios pesquisadores; posteriormente, foi avaliado por três especialistas da área para verificar a pertinência e adequação do instrumento; entretanto, não foi realizada uma avaliação psicométrica. Foi realizado um pré-teste com o instrumento em um município do interior de São Paulo; em seguida, os pesquisadores analisaram a presença de questões difíceis, ambíguas e mal formuladas, permitindo a melhoria do questionário para uma melhor compreensão das perguntas. Cabe destacar que o pré-teste foi aplicado a 49 participantes e os resultados foram incluídos na amostra final.

O instrumento de coleta supracitado foi construído com base no Protocolo de Manejo Clínico do Coronavírus (COVID-19) na APS do Ministério da Saúde^(17)^, contendo as seguintes variáveis: medidas para evitar contágio nas unidades de saúde; estratificação da gravidade da Síndrome Gripal; manejo terapêutico; encaminhamento a serviços de urgência/emergência ou hospitalares de casos graves; monitoramento clínico; medidas de prevenção comunitária e apoio à vigilância ativa.

A coleta de dados foi realizada por uma equipe composta por cinco coletadores que, inicialmente, encaminharam o link do questionário para e-mails disponíveis publicamente nas páginas oficiais na internet das secretarias municipais de saúde. Ainda, para aumentar a adesão, a pesquisa contou com a colaboração do Conselho Nacional de Secretários de Saúde (CONASS) e do Conselho Nacional de Secretários Municipais de Saúde (CONASEMS), que divulgaram e ressaltaram a importância da participação na pesquisa entre as secretarias de saúde. Além disso, os apoiadores dos Conselhos de Secretários Municipais de Saúde colaboraram na divulgação da pesquisa junto aos Departamentos Regionais de Saúde. Essa medida foi adotada para tentar alcançar o máximo de profissionais possíveis de todas as regiões brasileiras, garantindo a mesma chance de resposta a todos eles.

### Análise de dados

Para a análise da cobertura de eSF, não foi encontrado na literatura um parâmetro ideal sobre a cobertura desses serviços. Nesse contexto, os pesquisadores definiram a seguinte classificação: < 25%; de 25,01% a 49,99%; de 50% a 74,99% e >75%. A cobertura de eSF dos municípios participantes foi retirada do site e-Gestor AB do Ministério da Saúde^(18)^, sendo considerada a variável de exposição e, como variáveis de desfecho, foram consideradas as referentes à reorganização dos serviços da APS, sendo estas do tipo composto.

Inicialmente, os dados foram descritos por meio de frequências absolutas e percentuais (variáveis qualitativas) e por meio de medidas como média, desvio-padrão, mínimo, mediana e máximo (variáveis quantitativas).

Para estimar as razões de prevalência (RP) e intervalos de confiança de 95% (IC95%), comparando a faixa de cobertura de eSF, foi utilizado o modelo de regressão de Poisson com efeito aleatório^(19)^. Todas as análises foram realizadas por meio do *software* SAS 9.4. Para todas as análises, adotou-se um nível de significância de 5%.

### Aspectos éticos

A pesquisa foi aprovada pelo Comitê de Ética da Universidade Federal de São Carlos, CAAE 52527521.8.0000.5504.

## Resultados

Participaram da pesquisa 1474 gerentes de serviços de saúde da APS, procedentes de municípios de todo o país, destacando-se que 45,9% (676) eram da região Sudeste, 21% (311) do Nordeste, 17,5% (258) do Sul, 11,7% (173) do Centro-Oeste e 3,8% (56) do Norte. Ainda, 86,6% (1276) referiram ser do sexo feminino e 13,4% (198) do sexo masculino e apresentam idade média de 38,9 anos. O fato do estudo apresentar alta proporção de participantes da região Sudeste pode gerar viés de prevalência, entretanto, a amostra contém participantes de todos os estados e apresenta resultados consistentes e robustos sobre a reorganização da APS brasileira durante a fase crítica da pandemia.

Nas comparações por cobertura eSF, a adoção de medidas de prevenção de contágio nas unidades de saúde foi citada por todos os participantes. Dentre essas medidas, destaca-se que o distanciamento de um paciente a outro foi 10% (IC: 1,03; 1,17) mais prevalente em municípios com cobertura de eSF menor que 25% em comparação com municípios com cobertura entre 25% e 49,99%; 8% (IC: 1,03; 1,13) maior em comparação com municípios com cobertura entre 50% e 74,99%, e 9% (IC: 1,05; 1,14) maior em relação a municípios com cobertura maior ou igual a 75%. A realização da limpeza e desinfecção de equipamentos utilizados de um paciente a outro foi 12% (IC: 1,02; 1,22) mais prevalente em municípios com cobertura menor que 25% quando comparado com os de cobertura entre 50% e 74,99% (Tabela 1).

Todos os participantes afirmaram que o serviço de saúde possuía condições para atendimento de pacientes com suspeita ou diagnóstico de COVID-19 em ala separada dos outros, sendo que a prevalência foi 33% (IC: 1,04; 1,7) maior em municípios com cobertura de eSF menor que 25% quando comparado com municípios com cobertura entre 25% e 49,99% (Tabela 1).

**Tabela 1-:** Comparações entre os municípios, segundo cobertura de eSF*, quanto à adoção de medidas de prevenção de contágio em unidades de saúde. São Carlos, SP, Brasil, 2022

**Variável**	**Comparações**					
	**<25% vs entre 25% e 49,99%**		**<25% vs entre 50% e 74,99%**		**<25% vs >75%**	
	**RP** ^†^ **(IC 95%)** ^‡^	**Valor-p** ^§^	**RP** ^†^ **(IC 95%)** ^‡^	**Valor-p** ^§^	**RP** ^†^ **(IC 95%)** ^‡^	**Valor-p** ^§^
Considerando a possibilidade de o paciente (e acompanhante) estar contaminado com COVID-19, existem medidas de prevenção de contágio na Unidade de Saúde?						
Não	-	-	-	-	-	-
Sim	1 (1; 1)	-	1,01 (1; 1,01)	0,12	1 (1; 1,01)	0,16
Se sim, indique quais são: ^||^						
Identificação de sintomas (como tosse, coriza e dificuldade para respirar)	0,98 (0,92; 1,05)	0,60	0,97 (0,92; 1,03)	0,37	0,99 (0,93; 1,05)	0,68
Orientação e disponibilidade do uso de máscara cirúrgica.	1,04 (0,94; 1,14)	0,45	1,03 (0,96; 1,11)	0,37	1,05 (0,98; 1,13)	0,14
Distanciamento de um paciente a outro	1,1 (1,03; 1,17)	<0,01	1,08 (1,03; 1,13)	<0,01	1,09 (1,05; 1,14)	<0,01
Alas separadas para pacientes que indicaram presença de sintomas semelhantes aos da COVID-19	1,12 (0,81; 1,54)	0,50	0,83 (0,62; 1,12)	0,23	1,08 (0,85; 1,37)	0,52
Realização da limpeza e desinfecção de equipamentos utilizados de um paciente a outro.	1,07 (0,99; 1,16)	0,10	1,12 (1,02; 1,22)	0,01	1,05 (0,99; 1,12)	0,12
Há condições de atender pacientes com suspeita ou diagnóstico de COVID-19 em ala separada dos outros pacientes?						
Não	-	-	-	-	-	-
Sim	1,33 (1,04; 1,7)	0,02	0,97 (0,75; 1,26)	0,82	1,08 (0,89; 1,31)	0,44

*eSF = Equipe de saúde da família; ^†^RP = Razão de prevalência; ^‡^IC 95% = Intervalo de confiança; ^§^Valor-p = Nível de significância; ^||^É possível mais de uma resposta por respondente

Quanto às medidas preventivas de contágio adotadas nas unidades de saúde, quando as comparações foram realizadas em municípios com cobertura de eSF entre 25% e 49,99% e 50% e 74,99% e maior que 75%, a disponibilidade de alas separadas para pacientes que indicaram presença de sintomas semelhantes aos da COVID-19 foi 30% (IC: 1,05; 1,61) mais prevalente em municípios com cobertura entre 50% e 74,99% quando comparado à cobertura maior que 75% (Tabela 2).

Em relação às condições para atendimento de pacientes com suspeita ou diagnóstico de COVID-19 em ala separada dos outros pacientes, apresentou 27% (IC: 0,57; 0,94) menos prevalente em municípios com cobertura de eSF entre 25% e 49,99% do que em municípios com cobertura entre 50% e 74,99%; e na comparação entre municípios com cobertura 25% e 49,99% e maior que 75%, esta mesma variável foi 19% (0,68; 0,97) menos prevalente em municípios com menor cobertura (Tabela 2).

**Tabela 2-:** Comparações entre os municípios, segundo cobertura de eSF*, quanto à adoção de medidas preventivas de contágio em unidades de saúde. São Carlos, SP, Brasil, 2022

**Variável**	**Comparações**					
	**Entre 25% e 49,99% vs entre 50% e 74,99%**		**Entre 25% e 49,99% vs >75%**		**Entre 50% e 74,99% vs >75%**	
	**RP** ^†^ **(IC 95%)** ^‡^	**Valor-p** ^§^	**RP** ^†^ **(IC 95%)** ^‡^	**Valor-p** ^§^	**RP** ^†^ **(IC 95%)** ^‡^	**Valor-p** ^§^
Considerando a possibilidade de o paciente (e acompanhante) estar contaminado com COVID-19, existem medidas de prevenção de contágio na Unidade de Saúde?						
Não	-	-	-	-	-	-
Sim	1,01 (1; 1,01)	0,12	1 (1; 1,01)	0,16	1 (0,99; 1)	0,38
Se sim, indique quais são: ^||^						
Identificação de sintomas (como tosse, coriza e dificuldade para respirar).	0,99 (0,96; 1,03)	0,57	1 (0,97; 1,04)	0,78	1,02 (0,99; 1,04)	0,24
Orientação e disponibilidade do uso de máscara cirúrgica	1 (0,92; 1,08)	0,90	1,02 (0,94; 1,1)	0,70	1,02 (0,98; 1,06)	0,32
Distanciamento de um paciente a outro	0,98 (0,92; 1,05)	0,63	1 (0,94; 1,06)	0,91	1,01 (0,97; 1,06)	0,57
Alas separadas para pacientes que indicaram presença de sintomas semelhantes aos da COVID-19	0,75 (0,55; 1,01)	0,06	0,97 (0,76; 1,24)	0,79	1,3 (1,05; 1,61)	0,02
Realização da limpeza e desinfecção de equipamentos utilizados de um paciente a outro	1,04 (0,95; 1,14)	0,37	0,98 (0,92; 1,05)	0,56	0,94 (0,88; 1,01)	0,09
Há condições de atender pacientes com suspeita ou diagnóstico de COVID-19 em ala separada dos outros pacientes?						
Não	-	-	-	-	-	-
Sim	0,73 (0,57; 0,94)	0,01	0,81 (0,68; 0,97)	0,02	1,11 (0,91; 1,35)	0,29

*eSF = Equipe de saúde da família; ^†^RP = Razão de prevalência; ^‡^IC 95% = Intervalo de confiança; ^§^Valor-p = Nível de significância; ^||^É possível mais de uma resposta por respondente

Em relação às medidas adotadas no manejo de casos leves de COVID-19, a orientação de hidratação foi 8% (IC: 1,03; 1,14) mais prevalente em municípios com cobertura de eSF menor que 25% do que em municípios com cobertura entre 25% e 49,99%, e 6% (IC: 1,01; 1,1 e IC: 1,03; 1,1) mais prevalente do que em municípios com coberturas superiores a 50%. Já a orientação sobre alimentação foi 14% (IC: 1,06; 1,24) mais prevalente em municípios com cobertura menor que 25% do que em municípios com cobertura entre 25% e 49,99%; 9% (IC: 1,01; 1,16) mais prevalente em municípios com cobertura inferior a 25% quando comparado com cobertura maior que 75% (Tabela 3).

A orientação quanto ao isolamento domiciliar foi 7% (IC: 1,02; 1,12) mais prevalente em municípios com cobertura de eSF menor que 25% do que em municípios com cobertura entre 25% e 49,99%; 4% (IC:1,01; 1,07) mais prevalente em municípios com cobertura menor que 25% do que em coberturas maiores que 50%; o uso de analgésicos foi 15% (IC: 1,01; 1,31) mais prevalente em municípios com cobertura menor que 25% do que quando comparado com cobertura entre 25% e 49,99%. Já o teleatendimento para monitoramento de casos leves de COVID-19 foi 60% (IC: 1,21; 2,11) mais prevalente em municípios com cobertura menor que 25% do que em municípios com cobertura entre 25% e 49,99% (Tabela 3).

A realização de vigilância ativa e continuada de pacientes que foram acompanhados foi 21% (IC: 0,66; 0,93) menos prevalente em municípios com cobertura menor que 25% do que em municípios com cobertura maior que 75%; a revisão dos sintomas e o seguimento da evolução do quadro a cada 48 horas, preferencialmente por telefone, apresentou resultado semelhante, sendo 21% (IC: 0,63; 0,99) menos prevalente em municípios com cobertura menor que 25% do que em municípios com cobertura maior que 75% (Tabela 3).

Também se destaca que, na verificação da disponibilidade de leitos em hospital de referência para encaminhamento de pacientes que necessitavam de internação, antes de encaminhá-lo, a prevalência foi 32% (IC: 0,48; 0,96) menor em municípios com cobertura menor que 25% quando comparado com cobertura maior ou igual a 75% (Tabela 3).

**Tabela 3-:** Comparações entre os municípios, segundo cobertura de eSF*, quanto às medidas de manejo dos casos leves, monitoramento e vigilância ativa e continuada, verificação da disponibilidade de leitos antes de encaminhar. São Carlos, SP, Brasil, 2022

**Variável**	**Comparações**					
	**<25% vs entre 25% e 49,99%**		**<25% vs entre 50% e 74,99%**		**<25% vs >75%**	
	**RP** ^†^ **(IC 95%)** ^‡^	**Valor-p** ^§^	**RP** ^†^ **(IC 95%)** ^‡^	**Valor-p** ^§^	**RP** ^†^ **(IC 95%)** ^‡^	**Valor-p** ^§^
Quais medidas foram adotadas para o manejo dos casos leves de COVID-19? ^||^						
Orientação de repouso	1,03 (0,96; 1,1)	0,46	1 (0,95; 1,06)	0,92	1,02 (0,96; 1,07)	0,58
Orientação de hidratação	1,08 (1,03; 1,14)	<0,01	1,06 (1,01; 1,1)	0,01	1,06 (1,03; 1,1)	<0,01
Orientação de alimentação	1,14 (1,06; 1,24)	<0,01	1,06 (0,99; 1,15)	0,11	1,09 (1,01; 1,16)	0,02
Isolamento domiciliar	1,07 (1,02; 1,12)	<0,01	1,04 (1,01; 1,07)	0,02	1,04 (1,01; 1,07)	0,01
Analgésicos	1,15 (1,01; 1,31)	0,04	1 (0,9; 1,12)	0,94	1,08 (0,99; 1,17)	0,07
Antitérmicos	1,16 (1; 1,35)	0,05	1,03 (0,91; 1,17)	0,63	1,07 (0,98; 1,18)	0,13
Isolamento domiciliar por 14 dias a contar da data de início dos sintomas	0,94 (0,78; 1,13)	0,49	1,01 (0,81; 1,24)	0,96	1,02 (0,87; 1,21)	0,79
A unidade de saúde realizou teleatendimento para monitoramento de casos leves de COVID-19?						
Não	-	-	-	-	-	-
Sim	1,6 (1,21; 2,11)	<0,01	1,17 (0,94; 1,45)	0,16	1,15 (0,99; 1,34)	0,08
A unidade de saúde realiza vigilância ativa e continuada de pacientes que estão recebendo acompanhamento?						
Não	-	-	-	-	-	-
Sim	1,03 (0,8; 1,32)	0,81	0,86 (0,7; 1,07)	0,17	0,79 (0,66; 0,93)	<0,01
A unidade de saúde realiza a revisão dos sintomas e o seguimento da evolução do quadro a cada 48 horas, preferencialmente por telefone, solicitando consulta presencial quando necessário de exame físico?						
Não	-	-	-	-	-	-
Sim	1,16 (0,85; 1,58)	0,35	0,96 (0,69; 1,33)	0,79	0,79 (0,63; 0,99)	0,04
A unidade de saúde verifica se o hospital de referência para casos de internação por COVID-19 possui leitos suficientes e disponíveis para receber o paciente que necessita de internação, antes de encaminhá-lo?						
Não	-	-	-	-	-	-
Sim	1,23 (0,74; 2,04)	0,42	0,83 (0,56; 1,23)	0,35	0,68 (0,48; 0,96)	0,03

*eSF = Equipe de saúde da família; ^†^RP = Razão de prevalência; ^‡^IC 95% = Intervalo de confiança; ^§^Valor-p = Nível de significância; ^||^É possível mais de uma resposta por respondente

Ainda em relação às medidas adotadas no manejo de casos leves de COVID-19, a orientação de alimentação foi 7% (IC: 0,87; 0,99) menos prevalente em municípios com cobertura de eSF entre 25% e 49,99% do que em municípios entre 50% e 74,99%. O teleatendimento para monitoramento de casos leves de COVID-19 foi 27% (IC: 0,55; 0,98) menos prevalente em municípios com cobertura entre 25% e 49,99% do que em municípios com cobertura entre 50% e 74,99%; e 28% (IC: 0,56; 0,92) menos prevalente em municípios com cobertura entre 25% e 49,99% do que quando comparado à cobertura maior que 75% (Tabela 4).

A realização de vigilância ativa e continuada de pacientes que foram acompanhados foi 24% (IC: 0,62; 0,94) menos prevalente em municípios com cobertura de eSF entre 25% e 49,99% do que em municípios com cobertura maior que 75%; e a revisão dos sintomas e o seguimento da evolução do quadro a cada 48 horas, preferencialmente por telefone, foi 32% (IC: 0,54; 0,87) menor em municípios com cobertura de eSF entre 25% e 49,99% do que em municípios com cobertura maior que 75% (Tabela 4).

Já na verificação da disponibilidade de leitos em hospital de referência para encaminhamento de pacientes que necessitavam de internação, antes de encaminhá-lo, a prevalência foi 45% (IC: 0,38; 0,81) menor em municípios com cobertura entre 25% e 49,99% do que em municípios com cobertura maior que 75% (Tabela 4).

**Tabela 4-:** Comparações entre os municípios, segundo cobertura de eSF*, quanto às medidas de manejo dos casos leves, monitoramento e vigilância ativa e continuada, verificação da disponibilidade de leitos antes de encaminhar. São Carlos, SP, Brasil, 2022

**Variável**	**Comparações**					
	**Entre 25% e 49,99% vs entre 50% e 74,99%**		**Entre 25% e 49,99% vs >75%**		**Entre 50% e 74,99% vs >75%**	
	**RP** ^†^ **(IC 95%)** ^‡^	**Valor-p** ^§^	**RP** ^†^ **(IC 95%)** ^‡^	**Valor-p** ^§^	**RP** ^†^ **(IC 95%)** ^‡^	**Valor-p** ^§^
Quais medidas foram adotadas para o manejo dos casos leves de COVID-19? ^||^						
Orientação de repouso	0,98 (0,92; 1,04)	0,44	0,99 (0,94; 1,04)	0,68	1,01 (0,97; 1,05)	0,54
Orientação de hidratação	0,97 (0,93; 1,02)	0,25	0,98 (0,94; 1,02)	0,32	1,01 (0,97; 1,04)	0,72
Orientação de alimentação	0,93 (0,87; 0,99)	0,03	0,95 (0,9; 1,01)	0,08	1,02 (0,97; 1,08)	0,41
Isolamento domiciliar	0,97 (0,92; 1,01)	0,16	0,97 (0,92; 1,01)	0,17	1 (0,98; 1,03)	0,93
Analgésicos	0,88 (0,76; 1,01)	0,06	0,94 (0,84; 1,06)	0,32	1,08 (0,98; 1,18)	0,12
Antitérmicos	0,89 (0,76; 1,03)	0,12	0,92 (0,81; 1,05)	0,22	1,04 (0,94; 1,15)	0,41
Isolamento domiciliar por 14 dias a contar da data de início dos sintomas	1,07 (0,91; 1,26)	0,40	1,09 (0,99; 1,2)	0,08	1,02 (0,88; 1,17)	0,81
A unidade de saúde realizou teleatendimento para monitoramento de casos leves de COVID-19?						
Não	-	-	-	-	-	-
Sim	0,73 (0,55; 0,98)	0,04	0,72 (0,56; 0,92)	<0,01	0,98 (0,82; 1,18)	0,85
A unidade de saúde realiza vigilância ativa e continuada de pacientes que estão recebendo acompanhamento?						
Não	-	-	-	-	-	-
Sim	0,83 (0,65; 1,07)	0,15	0,76 (0,62; 0,94)	<0,01	0,91 (0,78; 1,07)	0,26
A unidade de saúde realiza a revisão dos sintomas e o seguimento da evolução do quadro a cada 48 horas, preferencialmente por telefone, solicitando consulta presencial quando necessário de exame físico?						
Não	-	-	-	-	-	-
Sim	0,82 (0,58; 1,16)	0,27	0,68 (0,54; 0,87)	<0,01	0,83 (0,63; 1,08)	0,17
A unidade de saúde verifica se o hospital de referência para casos de internação por COVID-19 possui leitos suficientes e disponíveis para receber o paciente que necessita de internação, antes de encaminhá-lo?						
Não	-	-	-	-	-	-
Sim	0,68 (0,44; 1,03)	0,07	0,55 (0,38; 0,81)	<0,01	0,82 (0,67; 1)	0,05

*eSF = Equipe de saúde da família; ^†^RP = Razão de prevalência; ^‡^IC 95% = Intervalo de confiança; ^§^Valor-p = Nível de significância; ^||^É possível mais de uma resposta por respondente

## Discussão

Os resultados deste estudo evidenciam que a cobertura de eSF influenciou a reorganização do processo de trabalho durante a fase crítica da pandemia de COVID-19. Municípios com coberturas de eSF menores que 25% apresentaram resultados de maior prevalência na implementação de medidas de prevenção e contágio da COVID-19, além da reorganização do manejo clínico de casos leves e do processo de trabalho.

Nesse contexto, municípios mais populosos tendem a ter menor cobertura de eSF que os menores. Essa cobertura reduzida em grandes centros se deve à maior complexidade e densidade populacional, dificultando a implementação e universalização da APS. No entanto, isso não indica falta de esforços, pois muitos compensam com maior variedade de serviços e redes mais complexas e robustas^(20)^.

Por outro lado, municípios pequenos tendem a ter maior cobertura de eSF, assim como áreas vulneráveis de municípios médios e grandes^(20)^, que, na fase crítica da pandemia, foram os mais impactados pelo avanço da doença. Nesse período, exigiram-se ações mais rápidas e efetivas da gestão pública para reorganizar fluxos na APS e encaminhar casos com agravamento clínico por COVID-19.

Os resultados deste estudo sugerem que municípios maiores, com menor cobertura de eSF, reorganizaram os serviços da APS de forma mais prática para otimizar os recursos disponíveis, geralmente com baixa densidade tecnológica e escassez de mão de obra. Nesse cenário, municípios paulistas com mais de 50 mil habitantes orientaram que todos os serviços de APS atendessem pacientes com COVID-19, com ou sem unidades exclusivas para o enfrentamento da doença^(21)^, impondo uma necessidade inerente de adaptação à realidade daquele momento àqueles profissionais. De forma semelhante, cidades da América Latina, especialmente no Chile e Colômbia, reorganizaram a assistência aos pacientes de COVID-19 em unidades próprias, mas também aproveitando a estrutura já existente da APS^(22)^.

Dentre as medidas adotadas para reorganizar as eSF, destacam-se ações de prevenção, realocação de alas para sintomáticos e reorganização do fluxo interno, que contribuíram para o controle da COVID-19. Na fase inicial, medidas como isolamento, quarentena, distanciamento físico e controle de fluxo foram eficazes na redução da contaminação, ajudando a achatar a curva de transmissão^(23)^. Países como Itália, França, Alemanha e Espanha, que adotaram *lockdown* rigoroso e ampla testagem, reduziram a letalidade e controlaram mutações virais. Em contraste, na Suécia, onde o *lockdown* foi mais brando, surgiram novas mutações do vírus^(24)^.

Como a COVID-19 é transmitida por via respiratória, especialmente em locais aglomerados, a reorganização do fluxo nos serviços de saúde foi essencial. Separar usuários com suspeita ou confirmação da doença dos demais reduziu o risco de transmissão. Essa reorganização também permitiu um manejo mais seguro, protegendo profissionais e usuários, e garantindo a continuidade dos serviços de forma segura e eficiente^(25)^. No Canadá, a rápida instalação de centros de isolamento nas unidades gerou bons resultados já no início da pandemia^(26)^. Na França, criaram-se áreas de espera e corredores específicos para sintomáticos nos serviços da APS^(16)^.

Ressalta-se que municípios com menor cobertura de eSF, maior porte e densidade populacional podem ter centralizado o atendimento a pessoas com sintomas e/ou COVID-19 em certos serviços, reorganizando as eSF para garantir a continuidade do cuidado e preservar integralidade, longitudinalidade e acesso durante a emergência sanitária. Além disso, esses municípios tinham maior capacidade de organizar os serviços em todos os níveis, com mais possibilidades de mobilizar equipamentos, suprimentos, pessoal e alternativas nas funções dos serviços de saúde^(27)^.

A emergência da COVID-19 exigiu uma ação rápida dos sistemas de saúde em âmbito global, que, em um primeiro momento, direcionaram a atenção para a garantia de leitos hospitalares à população^(28)^; mas, concomitante ou logo em seguida à reorganização do nível hospitalar, foi necessária a reestruturação imediata dos serviços da APS, uma vez que os hospitais ficaram sobrecarregados, a exemplo do colapso observado inicialmente na Itália^(29)^, e constatou-se que a maioria dos casos era considerada sem complexidade, o que possibilitaria uma capilarização do atendimento a partir dos serviços de APS, que até então permaneciam subutilizadas^(30)^.

Vale destacar que a APS deve ter alta resolutividade nas necessidades básicas de saúde e ser eficaz na redução de hospitalizações por condições sensíveis. No entanto, sua subutilização agravou, durante a pandemia, a crise já existente em um sistema historicamente subfinanciado, contribuindo para a escassez de recursos. A fragilidade dessa base comprometeu o monitoramento de casos de COVID-19 e a realização de ações efetivas de busca ativa e vigilância, aumentando as hospitalizações e sobrecarregando os serviços de emergência. O fortalecimento da APS poderia ter gerado uma resposta mais eficiente e mitigado o impacto da pandemia no Brasil^(28)^.

Os atributos da APS, nela incluída a eSF, como a adscrição do território, vínculo com o usuário e a longitudinalidade do cuidado, foram pouco explorados em muitos municípios brasileiros, principalmente no primeiro ano da pandemia. Durante a pandemia, foi verificada uma redução significativa na utilização de serviços de saúde preventivos e terapêuticos, sendo atribuída às restrições de mobilidade, distanciamento físico e ao medo da infecção^(31)^.

Apesar do atraso no redirecionamento da APS para o enfrentamento da pandemia, os serviços foram se adaptando e se fortalecendo, até assumirem papel central no atendimento durante a emergência sanitária. A capacidade de adaptação e inovação da APS permitiu reorganizar com eficiência o fluxo de usuários e gerenciar novos protocolos clínicos, reforçando a importância da qualificação da APS para garantir a continuidade do cuidado e a gestão de condições de saúde além da emergência^(32)^.

Durante a fase crítica da pandemia, a adaptação da APS e a intensificação do trabalho expuseram diferenças marcantes entre municípios, devido à ausência de diretrizes nacionais. Desigualdades locorregionais na estrutura da APS ― como recursos humanos, acesso a insumos, tecnologias e capacidade de coordenação ― acentuaram respostas desiguais à crise. Municípios com maior cobertura de eSF, gestão estruturada e integração com a vigilância epidemiológica mostraram melhor resposta, enquanto localidades com baixa densidade técnica ou fragilidades históricas enfrentaram mais dificuldades na organização do cuidado e manejo clínico da COVID-19. Essas diferenças se agravaram com a falta de protocolos unificados, forçando gestores e profissionais a tomarem decisões locais em meio a incertezas^(3,33)^.

Para além da gestão clínica, o manejo da dor e orientações sobre alimentação e ingesta hídrica adequadas não podem ser dissociados da educação em saúde, sendo este um dos pilares fundamentais da eSF. Na pandemia, a educação em saúde foi essencial não apenas para promover ações preventivas da doença, como o uso de máscaras e o distanciamento físico, mas também para combater a desinformação sobre o vírus e as vacinas, fornecer orientações sobre a identificação de sintomas e cuidados em casa, redução do estigma associado à infecção e oferta de apoio para a saúde mental^(34)^.

Cabe destacar que a eSF oferece uma assistência direcionada a grupos prioritários definidos por políticas públicas de saúde, crianças, pessoas idosas, mulheres, pessoas com hipertensão, diabetes e obesidade e saúde mental^(35)^. Nesse contexto, a COVID-19, considerada uma doença emergente e atualmente endêmica, deve ser incluída no arcabouço de atividades da eSF, ou seja, a gestão deve buscar meios para capacitar os profissionais de saúde frente a uma nova demanda para que possam oferecer uma assistência integral e resolutiva, além das ações de vigilância, independentemente da cobertura populacional do município.

Ainda, os resultados deste estudo evidenciaram que a vigilância de casos suspeitos ou confirmados foi mais frequente em localidades com maior cobertura de eSF. Uma razão para este êxito pode estar relacionada à composição das eSF, em especial à atuação dos Agentes Comunitários de Saúde (ACS). A atuação dos ACS durante a pandemia foi determinante para o sucesso da vigilância comunitária, uma vez que promoveram o cuidado territorializado e contínuo, mesmo em cenários adversos e de alta demanda, ampliando o alcance das ações de vigilância, especialmente em áreas vulneráveis com acesso restrito aos serviços de saúde^(36-37)^.

A eficácia do trabalho das equipes de eSF na vigilância ativa de casos de COVID-19, e até mesmo a própria assistência direcionada a esses usuários, esteve associada à utilização de tecnologias de comunicação e informação, como telemedicina e plataformas digitais, o que facilitou as consultas virtuais, o monitoramento remoto e a disseminação de informações, aumentando a acessibilidade e eficiência dos cuidados. Essas ações integradas foram fundamentais para controlar a disseminação do vírus e garantir a continuidade do cuidado^(38)^. Municípios com uma maior cobertura de eSF são, em sua maioria, de médio e pequeno porte, que podem muitas vezes, serem serviços únicos de saúde no município, possuem um maior controle e eficácia das ações de vigilância, devido ao acesso a muitos domicílios, vínculo com a população e demanda por serviços. Assim, essa característica pode justificar a maior adesão desses serviços de saúde às ações de vigilância, acompanhamento da evolução do quadro clínico do usuário e utilização de telemedicina e telemonitoramento.

As ações efetivas de vigilância em saúde, promovidas com maior prevalência nos municípios com ampla cobertura da eSF, não apenas favoreceram o monitoramento e o controle da COVID-19, mas também contribuíram para o encaminhamento adequado de casos graves, otimizando a utilização dos leitos hospitalares disponíveis. Nesse contexto, destaca-se a importância da articulação entre os diferentes pontos da Rede de Atenção à Saúde (RAS), com ênfase na integração entre a APS e os serviços de média e alta complexidade, uma vez que o fluxo contínuo de informações, aliado ao treinamento e capacitação dos profissionais de saúde, mostrou-se essencial para o aproveitamento eficaz dos recursos existentes, sobretudo em situações de escassez^(39)^. Ainda, em um outro cenário, na Noruega, foi verificada uma integração entre serviços da APS e outros serviços de saúde, por meio de protocolos e diretrizes unificadas, de forma a manter um atendimento seguro a todos pacientes^(40)^.

Entretanto, a pandemia agravou as desigualdades existentes na distribuição de leitos hospitalares entre os municípios brasileiros, pois regiões com menores condições socioeconômicas enfrentaram maiores dificuldades de acesso à hospitalização, resultando em maiores coeficientes de internação tardia e mortalidade. A centralização dos recursos hospitalares nas capitais e em cidades de médio porte dificultou o acesso oportuno aos cuidados intensivos para populações residentes em áreas remotas ou periféricas. Essa situação expôs fragilidades estruturais históricas do Sistema Único de Saúde (SUS) e evidenciou a urgência de uma redistribuição mais equitativa dos recursos de saúde, especialmente no que se refere à infraestrutura hospitalar^(41)^.

Diante desse cenário, torna-se imprescindível o fortalecimento da APS, não apenas como porta de entrada do sistema, mas também como coordenadora do cuidado, com capacidade resolutiva ampliada e estratégias de manejo clínico eficazes. A atuação qualificada das equipes da eSF, articulada a protocolos clínicos bem definidos, pode contribuir para a triagem precisa dos casos, garantir que os encaminhamentos para o nível hospitalar ocorram de forma oportuna, racional e com base na gravidade, contribuindo para a redução da sobrecarga dos hospitais e desfechos adversos^(42-43)^.

Por fim, os resultados deste estudo mostram que a presença de equipes de eSF permitiu uma resposta ágil e adaptativa, facilitando o manejo de pacientes com COVID-19, a promoção de medidas preventivas e o uso de tecnologias de comunicação, como a telemedicina. Ressalta-se que estas tecnologias permaneceram de forma significativa após a pandemia, especialmente em locais onde há maior maturidade digital, formação em saúde digital e infraestrutura favorável, sendo um grande legado, não só no Brasil como no mundo^(44)^.

Assim como no Brasil, a pandemia acelerou transformações na APS no Canadá, impulsionando a expansão de iniciativas digitais, como agendamento online e serviços emergenciais, como clínicas especializadas de COVID-19, além do teleatendimento. Essas mudanças deixaram um legado no que se refere à consolidação da telessaúde e à necessidade de modelos mais ágeis e integrados, capazes de responder a crises sem comprometer o acesso ao cuidado^(45)^. Ambos os casos reforçam o papel estratégico da APS e da infraestrutura digital em cenários de emergência sanitária.

O presente estudo apresenta limitações inerentes ao seu desenho transversal, como a ausência de causalidade e o viés de prevalência. A predominância de municípios da região Sudeste na amostra pode ter introduzido um viés de prevalência. Além disso, o uso de formulários autorrespondidos para a coleta de dados pode ter causado viés de resposta, devido à possível interpretação inadequada das perguntas e superestimação ou subestimação das informações fornecidas pelos participantes. Contudo, a amostra inclui representantes de todos os estados e oferece resultados consistentes e robustos sobre a reorganização da APS brasileira durante a fase crítica da pandemia, especialmente em relação à cobertura das eSF.

## Conclusão

A eSF desempenhou um papel essencial durante a fase crítica da pandemia de COVID-19 ao garantir um suporte contínuo e eficaz à população. O desenvolvimento de ações preventivas, monitoramento de casos leves e orientação comunitária contribuiu para a redução da disseminação do vírus e para a gestão das necessidades de saúde da população. Além disso, a eSF ajudou a manter a continuidade dos cuidados para condições crônicas e outras demandas de saúde, demonstrando sua importância na resposta a emergências sanitárias de forma adaptável e sustentável.

Sugere-se a realização de futuras pesquisas que avaliem o impacto da reorganização da APS, durante a pandemia, a longo prazo no processo de trabalho das equipes. Ainda, os resultados emergem a importância da criação e padronização de protocolos que orientem a reorganização dos serviços de saúde em situações de emergências em saúde pública.

# Translation: Cobertura de Salud de la Familia y reorganización de la asistencia provista durante la pandemia de COVID-19*

**DOI:** 10.1590/1518-8345.7726.4788

**Authors:** Rodrigo das Neves Cano, Ana Paula de Vechi Corrêa, Silvia Carla da Silva André Uehara

**Affiliations:** 1 Universidade Federal de São Carlos, São Carlos, SP, Brasil

**Keywords:** COVID-19, Pandemias, Atención Primaria de Salud, Vigilancia en Salud Pública, Gestión en Salud, Estrategias de Salud Nacionales

## Abstract

evaluar cómo se reorganizó la asistencia provista en el nivel de Atención Primaria de la Salud a personas con sospecha y/o confirmación de diagnóstico de COVID-19 durante la fase crítica de la pandemia, considerando la cobertura de los equipos de Salud de la Familia.

estudio transversal y analítico realizado con 1474 gerentes de servicios de Atención Primaria. Los datos se recolectaron en *Google Forms* y fueron analizados por medio de razones de prevalencia, utilizando el modelo de regresión de Poisson con efecto aleatorio.

los municipios con niveles de cobertura inferiores al 25% presentaron una prevalencia 10% superior en cuanto a la distancia entre pacientes, 33% superior en la atención de pacientes con sospecha o confirmación de diagnóstico de COVID-19 en un sector aparte, 60% superior en el uso de Teleasistencia para monitorear casos leves y 7% superior en términos de orientación sobre aislamiento domiciliario, en comparación con los municipios en los que los niveles de cobertura fueron del 25% al 49,99%

la asistencia provista en Atención Primaria de la Salud se reorganizó de distintas maneras entre cada Estrategia de Salud de la Familia: los municipios con niveles de cobertura más bajos implementaron medidas de prevención y monitoreo con mayor frecuencia. Este dato puntual indica la necesidad de diseñar y estandarizar protocolos que orienten la reorganización de los servicios de salud en situaciones de emergencia sanitaria.

## Destacados

**(1)** La cobertura de Salud de la Familia ejerció cierta influencia sobre la asistencia provista durante la pandemia. **(2)** Cuando los niveles de cobertura de Salud de la Familia fueron menores, se obtuvieron mejores resultados. **(3)** Se adoptaron medidas de prevención de forma más prevalente en niveles de cobertura inferiores al 25%. **(4)** El monitoreo por Teleasistencia fue más prevalente en niveles de cobertura inferiores al 25%. **(5)** La vigilancia activa y continua fue menos prevalente en niveles de cobertura inferiores al 25%.

## Introducción

La pandemia de COVID-19 produjo cambios económicos, políticos y sociales en diversos países^(1)^. Dichos cambios contribuyeron (y siguen haciéndolo) a evidenciar las fragilidades de los sistemas de salud en todo el mundo, que cabe destacar ya afrontaban dificultades antes de la pandemia^(2)^.

A nivel global, la Atención Primaria de la Salud (APS) fue esencial durante el proceso para readecuar los sistemas de salud a fin de hacer frente a la emergencia sanitaria causada por el COVID-19. Tanto el rol de la APS como ingreso al sistema como la descentralización de los sistemas de atención de la salud, la activa participación en la vigilancia sanitaria y el uso de tecnologías de la información y la comunicación fueron los puntos clave para una acción efectiva durante la pandemia; sin embargo, este nivel de asistencia se reorganizó de distintas formas y con distintas intensidades en cada localidad^(3)^.

Desde el comienzo de la pandemia, los servicios de salud debieron implementar cambios en sus instalaciones físicas, como así también en todo su proceso de trabajo y gestión, especialmente en la APS. La preocupación por garantizar ambientes seguros para que las personas pudieran circular y seguir controlando a los pacientes con patologías crónicas, además de dar continuidad a diversos programas en las áreas prioritarios, llevó a los servicios de APS a repensar sus formas de trabajo^(4-5)^.

En el ámbito mundial, la pandemia impuso sobre la APS la necesidad de adaptar el servicio para incorporar acciones de vigilancia y monitoreo específicas para el COVID-19, además de tratamientos domiciliarios intensificados, incluyendo Telemedicina o Telemonitoreo, muy utilizado en países como Israel, España e incluso Brasil^(6-8)^. En muchas localidades, el Telemonitoreo en APS fue importante desde el punto de vista estratégico tanto para monitorear pacientes durante el período de aislamiento como para estimular las medidas de asistencia en pacientes con mayor riesgo de infección^(9-10)^.

Como principal estrategia del sistema de salud vigente para fortalecer la APS, cabe señalar que la Estrategia de Salud de la Familia (ESF) se destacó en Brasil en el control del flujo de pacientes en las unidades, en la realización de pruebas de diagnóstico para COVID-19 y en la distribución de medicamentos, documentos y pautas orientativas en las unidades y en los domicilios, además de acciones para fomentar el aislamiento y distanciamiento físico. Además, el Telemonitoreo y las acciones de educación para la salud en la ESF demostraron ser prácticas importantes desde el comienzo de la pandemia, especialmente debido a las características de este servicio como su proximidad y vínculo con la población inscrita, viabilizando un monitoreo más preciso de los casos^(6,11-12)^.

Resulta cada vez más imperioso que los gerentes presten atención a las necesidades de la APS, tanto proporcionando infraestructura básica para realizar diversas actividades como manteniendo debidamente capacitado a su personal, especialmente durante emergencias sanitarias. Comprender las características inherentes a la dinámica de los cambios que impuso la pandemia representa un importante elemento que favoreció la reorganización de la APS en Brasil. También cabe destacar la ausencia de estudios comparativos sobre la reorganización de la APS conforme a distintos niveles de cobertura de la ESF durante la fase crítica de la pandemia.

En este contexto y tanto en Brasil como en otros países, la literatura demuestra cómo se fue organizando la APS durante la pandemia^(6,11-16)^. Sin embargo, se destaca que la mayoría de estos estudios se realizó durante el primer año de la pandemia, connotando el sentido de una fotografía; en oposición, este estudio se asemeja a una película, es decir, es un análisis longitudinal de la fase crítica de la pandemia. Por consiguiente, presenta resultados de vanguardia con notoriedad en el período posterior a la pandemia, puesto que contribuye directamente a llenar el vacío de conocimientos con respecto a la forma en la que se reorganizaron los servicios de la APS desde los aspectos estructural, asistencial y de vigilancia, según la perspectiva de los gerentes de dichos servicios y de forma comparativa, considerando los niveles de cobertura de la ESF y proporcionando evidencias científicas que fundamenten la toma de decisiones y la planificación en salud, especialmente para nuevas situaciones de emergencia sanitaria.

En consecuencia, el objetivo de este estudio fue evaluar cómo se reorganizó la asistencia provista en el nivel de Atención Primaria de la Salud a personas con sospecha y/o confirmación de diagnóstico de COVID-19 durante la fase crítica de la pandemia, considerando la cobertura de la ESF.

## Método

### Diseño del estudio

Estudio de corte transversal y analítico, redactado conforme a la herramienta *Strengthening the Reporting of Observational Studies in Epidemiology*(STROBE).

### Población y muestra

La población objetivo de este estudio estuvo compuesta por gerentes de servicios de APS de Brasil. Cabe destacar que debido a la ausencia de datos oficiales sobre la cantidad de gerentes actuantes en estos servicios, fue necesario recurrir a muestreo por conveniencia. En este contexto, la base de datos contó inicialmente con 1651 participantes, excluyendo respuestas duplicadas (n=30), respuestas incompletas (n=14) y formularios respondidos por profesionales que no eran gerentes de servicios de APS (n=133), conformando una muestra final de 1474 gerentes de servicios de salud de APS de municipios brasileños.

Se adoptó el siguiente criterio de inclusión: profesionales que hubieren estado a cargo de servicios de APS por al menos 3 meses durante la fase de emergencia sanitaria impuesta por la pandemia de COVID-19; a su vez, el criterio de exclusión correspondió a formularios incompletos.

### Recolección de datos

Los datos se recolectaron de forma remota debido al período de la pandemia de COVID-19 y dada la gran cantidad de participantes, entre abril y septiembre de 2022 y por medio de un cuestionario de auto-respuesta en *Google Forms*. El extenso período de recolección de datos se justifica por la dificultad para llegar a participantes de todas las regiones.

El cuestionario fue elaborado por los propios investigadores; posteriormente, tres especialistas del área lo evaluaron para verificar su pertinencia y adecuación. Sin embargo, no se realizó ninguna evaluación psicométrica. Se implementó una prueba previa con el instrumento en un municipio del interior de São Paulo; subsiguientemente, los investigadores analizaron la presencia de preguntas difíciles, ambiguas y mal formuladas, lo que permitió mejorar el nivel de comprensión de las preguntas. Cabe destacar que la prueba previa se aplicó a 49 participantes y que los resultados se incluyeron en la muestra final.

El instrumento para recolectar los datos antes mencionado se construyó sobre la base del Protocolo de Manejo Clínico del Coronavirus (COVID-19) en la APS del Ministerio de Salud^(17)^ e incluyó las siguientes variables: medidas para evitar el contagio en unidades de salud; estratificación de la gravedad del Síndrome gripal; manejo terapéutico; derivación de casos graves a servicios de urgencia/emergencia u hospitalarios; monitoreo clínico; y medidas de prevención comunitaria y apoyo a la vigilancia activa.

La recolección de datos estuvo a cargo de un equipo conformado por cinco personas que, como primer paso, reenviaron el enlace del cuestionario a direcciones de correo electrónico disponibles públicamente en las páginas oficiales de Internet correspondientes a las secretarías municipales de salud. Además, a fin de mejorar la adhesión, el trabajo de investigación contó con la colaboración del Consejo Nacional de Secretarios de Salud (CONASEMS) y del Consejo Nacional de Secretarios Municipales de Salud (CONASS), que anunciaron la investigación entre las secretarías de salud y destacaron la importancia de participar en ella. Adicionalmente, los colaboradores de los Consejos de Secretarios Municipales de Salud ayudaron a divulgar la investigación junto con los Departamentos Regionales de Salud. Tal medida se adoptó para tratar de llegar a la mayor cantidad posible de profesionales de todas las regiones de Brasil, garantizando así que todos tuvieran las mismas oportunidades de responder.

### Análisis de los datos

En la literatura no se encontró ningún parámetro ideal sobre la cobertura de estos servicios a fin de analizar la cobertura de la ESF. En este contexto, los investigadores definieron la siguiente clasificación: <25%; del 25,01% al 49,99%; del 50% al 74,99% y >75%. La cobertura de la ESF correspondiente a los municipios participantes se obtuvo del sitio *e-Gestor AB* del Ministerio de Salud^(18)^ y se la consideró como variable de exposición; a su vez, las variables referentes a la reorganización de los servicios de la APS (del tipo compuesto) se consideraron como las de desenlace.

Inicialmente, los datos se describieron por medio de frecuencias absolutas y porcentuales (variables cualitativas) y a través de medidas como valor medio, desviación estándar, mínimo, mediana y máximo (variables cuantitativas).

Para estimar las razones de prevalencia (RP) y los intervalos de confianza del 95% (IC95%) (comparando el rango de cobertura de la ESF), se utilizó el modelo de regresión de Poisson con efecto aleatorio^(19)^. Todos los análisis se realizaron en el programa de *software* SAS 9.4. Se adoptó un nivel de significancia del 5% para todos los análisis.

### Aspectos éticos

El trabajo de investigación fue aprobado por el Comité de Ética de la *Universidade Federal de São Carlos*, CAAE 52527521.8.0000.5504.

## Resultados

Los participantes de la investigación fueron 1474 gerentes a cargo de servicios de salud de la APS, procedentes de municipios de todo el país y destacando que el 45,9% (676), 21% (311), 17,5% (258), 11,7% (173) y 3,8% (56) era de las regiones Sudeste, Noreste, Sur, Centro-Oeste y Norte, respectivamente. Además, el 86,6% (1276) y el 13,4% (198) declaró pertenecer a los sexos femenino y masculino, respectivamente, con una media de edad de 38,9 años. El hecho de que el estudio presente una elevada proporción de participantes de la región Sudeste puede generar cierto sesgo de prevalencia; no obstante, la muestra incluye participantes de todos los estados y presenta resultados uniformes y sólidos sobre cómo se reorganizó la APS de Brasil durante la fase crítica de la pandemia.

En las comparaciones por niveles de cobertura de la ESF, todos los participantes citaron la adopción de medidas para prevenir el contagio en unidades de salud. Entre estas medidas, se destaca que la distancia entre pacientes fue un 10% (IC: 1,03; 1,17) más prevalente en municipios con niveles de cobertura de la ESF inferiores al 25% en comparación con aquellos en los que dichos niveles fueron del 25% al 49,99%, 8% (IC: 1,03; 1,13) más alto que en los municipios con niveles de cobertura del 50% al 74,99% y 9% (IC: 1,05; 1,14) superior en relación a municipios con niveles de cobertura mayores o iguales que 75%. La implementación de procedimientos de limpieza y desinfección de equipamientos utilizados de un paciente a otro fue un 12% (IC: 1,02; 1,22) más prevalente en municipios con niveles de cobertura inferiores al 25% en comparación con aquellos en los que dichos niveles eran del 50% al 74,99% (Tabla 1).

Todos los participantes afirmaron que el servicio de salud poseía condiciones aptas para atender pacientes con sospecha o confirmación de diagnóstico de COVID-19 en un ala aparte de los demás, con una prevalencia un 33% (IC: 1,04; 1,7) más alta en municipios con niveles de cobertura de la ESF inferiores al 25% en comparación con aquellos en los que dichos niveles eran del 25% al 49,99% (Tabla 1).

**Tabla 1-:** Comparaciones entre los municipios según niveles de cobertura de la ESF*, con respecto a la adopción de medidas para prevenir el contagio en unidades de salud. São Carlos, SP, Brasil, 2022

**Variable**	**Comparaciones**					
	**<25% vs 25%-49,99%**		**<25% vs 50%-74,99%**		**<25% vs >75%**	
	**RP** ^†^ **(IC95%)** ^‡^	**Valor p** ^§^	**RP** ^†^ **(IC95%)** ^‡^	**Valor p** ^§^	**RP** ^†^ **(IC95%)** ^‡^	**Valor p** ^§^
Considerando la posibilidad de que el paciente (y su acompañante) estuvieran contaminados con COVID-19, ¿hay medidas para prevenir el contagio implementadas en la Unidad de Salud?						
No	-	-	-	-	-	-
Sí	1 (1; 1)	-	1,01 (1; 1,01)	0,12	1 (1; 1,01)	0,16
De ser así, indicar cuáles son: ^||^						
Identificación de síntomas (como tos, secreción nasal y dificultad para respirar)	0,98 (0,92; 1,05)	0,60	0,97 (0,92; 1,03)	0,37	0,99 (0,93; 1,05)	0,68
Pautas orientativas sobre cómo usar mascarillas quirúrgicas y su disponibilidad	1,04 (0,94; 1,14)	0,45	1,03 (0,96; 1,11)	0,37	1,05 (0,98; 1,13)	0,14
Distancia entre pacientes	1,1 (1,03; 1,17)	<0,01	1,08 (1,03; 1,13)	<0,01	1,09 (1,05; 1,14)	<0,01
Alas aparte para pacientes que señalaron presencia de síntomas semejantes a los del COVID-19	1,12 (0,81; 1,54)	0,50	0,83 (0,62; 1,12)	0,23	1,08 (0,85; 1,37)	0,52
Procedimientos de limpieza y desinfección de equipamientos utilizados de un paciente a otro	1,07 (0,99; 1,16)	0,10	1,12 (1,02; 1,22)	0,01	1,05 (0,99; 1,12)	0,12
¿Están dadas las condiciones para atender pacientes con sospecha o confirmación de diagnóstico de COVID-19 en un ala aparte de los demás pacientes?						
No	-	-	-	-	-	-
Sí	1,33 (1,04; 1,7)	0,02	0,97 (0,75; 1,26)	0,82	1,08 (0,89; 1,31)	0,44

*ESF = Estrategia de Salud de la Familia; ^†^RP = Razón de Prevalencia; ^‡^IC95% = Intervalo de Confianza del 95%; ^§^Valor p = Nivel de significancia; ^||^Es posible que una misma persona haya dado más de una respuesta

En relación con las medidas para prevenir el contagio adoptadas en las unidades de salud, cuando se realizaron las debidas comparaciones entre municipios con niveles de cobertura de la ESF del 25% al 49,99%, del 50% al 74,99% y superiores al 75%, la disponibilidad de alas aparte para pacientes que señalaron presencia de síntomas semejantes a los del COVID-19 fue un 30% (IC: 1,05; 1,61) más prevalente en municipios con niveles del 50% al 74,99% en comparación con aquellos en los que la cobertura era superior al 75% (Tabla 2).

Con respecto a las condiciones para atender pacientes con sospecha o confirmación de diagnóstico de COVID-19 en un ala aparte de los demás pacientes, este aspecto se presentó un 27% (IC: 0,57; 0,94) menos prevalente en municipios con niveles de cobertura de la ESF del 25% al 49,99% en comparación con aquellos en los que dichos niveles eran del 50% al 74,99%. A su vez, al comparar municipios con niveles de cobertura del 25% al 49,99% con aquellos en los que la cobertura supera el 75%, esta misma variable fue un 19% ( IC: 0,68; 0,97) menos prevalente en municipios con menor cobertura (Tabla 2).

**Tabla 2-:** Comparaciones entre los municipios según niveles de cobertura de la ESF*, con respecto a la adopción de medidas para prevenir el contagio en unidades de salud. São Carlos, SP, Brasil, 2022

**Variable**	**Comparaciones**					
	**25%-49,99% vs 50%-74,99%**		**25%-49,99% vs >75%**		**50%-74,99% vs >75%**	
	**RP** ^†^ **(IC95%)** ^‡^	**Valor p** ^§^	**RP** ^†^ **(IC95%)** ^‡^	**Valor p** ^§^	**RP** ^†^ **(IC95%)** ^‡^	**Valor p** ^§^
Considerando la posibilidad de que el paciente (y su acompañante) estuvieran contaminados con COVID-19, ¿hay medidas para prevenir el contagio implementadas en la Unidad de Salud?						
No	-	-	-	-	-	-
Sí	1,01 (1; 1,01)	0,12	1 (1; 1,01)	0,16	1 (0,99; 1)	0,38
De ser así, indicar cuáles son: ^||^						
Identificación de síntomas (como tos, secreción nasal y dificultad para respirar)	0,99 (0,96; 1,03)	0,57	1 (0,97; 1,04)	0,78	1,02 (0,99; 1,04)	0,24
Pautas orientativas sobre cómo usar mascarillas quirúrgicas y su disponibilidad	1 (0,92; 1,08)	0,90	1,02 (0,94; 1,1)	0,70	1,02 (0,98; 1,06)	0,32
Distancia entre pacientes	0,98 (0,92; 1,05)	0,63	1 (0,94; 1,06)	0,91	1,01 (0,97; 1,06)	0,57
Alas aparte para pacientes que señalaron presencia de síntomas semejantes a los del COVID-19	0,75 (0,55; 1,01)	0,06	0,97 (0,76; 1,24)	0,79	1,3 (1,05; 1,61)	0,02
Procedimientos de limpieza y desinfección de equipamientos utilizados de un paciente a otro	1,04 (0,95; 1,14)	0,37	0,98 (0,92; 1,05)	0,56	0,94 (0,88; 1,01)	0,09
¿Están dadas las condiciones para atender pacientes con sospecha o confirmación de diagnóstico de COVID-19 en un ala aparte de los demás pacientes?						
No	-	-	-	-	-	-
Sí	0,73 (0,57; 0,94)	0,01	0,81 (0,68; 0,97)	0,02	1,11 (0,91; 1,35)	0,29

*ESF = Estrategia de Salud de la Familia; ^†^RP = Razón de Prevalencia; ^‡^IC95% = Intervalo de Confianza del 95%; ^§^Valor p = Nivel de significancia; ^||^Es posible que una misma persona haya dado más de una respuesta

En relación con las medidas adoptadas en el manejo de casos leves de COVID-19, la pauta de hidratación fue un 8% (IC: 1,03; 1,14) más prevalente en municipios con niveles de cobertura de la ESF inferiores al 25% que en aquellos en los que dichos niveles eran del 25% al 49,99% y un 6% (IC: 1,01; 1,1 e IC: 1,03; 1,1) más prevalente que en los municipios con niveles de cobertura superiores al 50%. A su vez, la pauta sobre alimentación fue un 14% (IC: 1,06; 1,24) más prevalente en municipios con niveles de cobertura inferiores al 25% que en aquellos en los que dichos niveles eran del 25% al 49,99% y 9% (IC: 1,01; 1,16) más prevalente en municipios con cobertura inferior al 25% en comparación con niveles de cobertura superiores al 75% (Tabla 3).

La pauta referente al aislamiento domiciliario fue un 7% (IC: 1,02; 1,12) más prevalente en municipios con niveles de cobertura de la ESF inferiores al 25% que en aquellos en los que dichos niveles eran del 25% al 49,99% y un 4% (IC: 1,01; 1,07) más prevalente en municipios con niveles de cobertura inferiores al 25% que en aquellos con niveles superiores al 50%. A su vez, el uso de analgésicos fue un 15% (IC: 1,01; 1,31) más prevalente en municipios con niveles de cobertura inferiores al 25% en comparación con aquellos en los que dichos niveles eran del 25% al 49,99%. En paralelo, la Teleasistencia para monitorear casos leves de COVID-19 fue un 60% (IC: 1,21; 2,11) más prevalente en municipios con niveles de cobertura inferiores al 25% que en aquellos en los que dichos niveles eran del 25% al 49,99 (Tabla 3).

La implementación de procedimientos de vigilancia activa y continua de pacientes sometidos a seguimiento fue un 21% (IC: 0,66; 0,93) menos prevalente en municipios con niveles de cobertura inferiores al 25% que en aquellos en los que dichos niveles superaban el 75%. Revisar los síntomas y seguir la evolución del cuadro clínico cada 48 horas (preferentemente por teléfono) presentó un resultado similar: 21% (IC: 0,63; 0,99) menos prevalente en municipios con niveles de cobertura inferiores al 25% que en aquellos en los que dichos niveles eran superiores al 75% (Tabla 3).

También se destaca que, al verificar la disponibilidad de camas en el hospital de referencia para derivar pacientes que precisaban internación, la prevalencia antes de dichas derivaciones fue un 32% (IC: 0,48; 0,96) menor en municipios con niveles de cobertura inferiores al 25% en comparación con niveles cobertura de al menos el 75% (Tabla 3).

**Tabla 3-:** Comparaciones entre los municipios según cobertura de la ESF*, con respecto a las medidas para manejar casos leves, al monitoreo y la vigilancia activa y continua, y a verificar la disponibilidad de camas antes de derivar pacientes. São Carlos, SP, Brasil, 2022

**Variable**	**Comparaciones**					
	**<25% vs 25%-49,99%**		**<25% vs 50%-74,99%**		**<25% vs >75%**	
	**RP** ^†^ **(IC95%)** ^‡^	**Valor p** ^§^	**RP** ^†^ **(IC95%)** ^‡^	**Valor p** ^§^	**RP** ^†^ **(IC95%)** ^‡^	**Valor p** ^§^
¿Qué medidas se adoptaron para manejar los casos leves de COVID-19? ^||^						
Pauta de reposo	1,03 (0,96; 1,1)	0,46	1 (0,95; 1,06)	0,92	1,02 (0,96; 1,07)	0,58
Pauta de hidratación	1,08 (1,03; 1,14)	<0,01	1,06 (1,01; 1,1)	0,01	1,06 (1,03; 1,1)	<0,01
Pauta de alimentación	1,14 (1,06; 1,24)	<0,01	1,06 (0,99; 1,15)	0,11	1,09 (1,01; 1,16)	0,02
Aislamiento domiciliario	1,07 (1,02; 1,12)	<0,01	1,04 (1,01; 1,07)	0,02	1,04 (1,01; 1,07)	0,01
Analgésicos	1,15 (1,01; 1,31)	0,04	1 (0,9; 1,12)	0,94	1,08 (0,99; 1,17)	0,07
Antipiréticos	1,16 (1; 1,35)	0,05	1,03 (0,91; 1,17)	0,63	1,07 (0,98; 1,18)	0,13
Aislamiento domiciliario por 14 días a partir de la fecha en la que se iniciaron los síntomas	0,94 (0,78; 1,13)	0,49	1,01 (0,81; 1,24)	0,96	1,02 (0,87; 1,21)	0,79
¿La unidad de salud ofreció Teleasistencia para monitorear casos leves de COVID-19?						
No	-	-	-	-	-	-
Sí	1,6 (1,21; 2,11)	<0,01	1,17 (0,94; 1,45)	0,16	1,15 (0,99; 1,34)	0,08
¿La unidad de salud implementa procedimientos de vigilancia activa y continua en el caso de pacientes sometidos a seguimiento?						
No	-	-	-	-	-	-
Sí	1,03 (0,8; 1,32)	0,81	0,86 (0,7; 1,07)	0,17	0,79 (0,66; 0,93)	<0,01
¿La unidad de salud revisa los síntomas y sigue la evolución del cuadro clínico cada 48 horas (preferentemente por teléfono), solicitando consultas presenciales cuando es necesario realizar un examen físico?						
No	-	-	-	-	-	-
Sí	1,16 (0,85; 1,58)	0,35	0,96 (0,69; 1,33)	0,79	0,79 (0,63; 0,99)	0,04
¿La unidad de salud verifica si el hospital de referencia para casos de internación por COVID-19 tiene suficientes camas disponibles para recibir a los pacientes que precisan ser hospitalizados antes de derivarlos?						
No	-	-	-	-	-	-
Sí	1,23 (0,74; 2,04)	0,42	0,83 (0,56; 1,23)	0,35	0,68 (0,48; 0,96)	0,03

*ESF = Estrategia de Salud de la Familia; ^†^RP = Razón de Prevalencia; ^‡^IC95% = Intervalo de Confianza del 95%; ^§^Valor p = Nivel de significancia; ^||^Es posible que una misma persona haya dado más de una respuesta

También en relación con las medidas adoptadas para manejar casos leves de COVID-19, la pauta de alimentación fue un 7% (IC: 0,87; 0,99) menos prevalente en municipios con niveles de cobertura de la ESF del 25% al 49,99% que en aquellos en los que dichos niveles eran del 50% al 74,99%. La Teleasistencia para monitorear casos leves de COVID-19 fue un 27% (IC: 0,55; 0,98) menos prevalente en municipios con niveles de cobertura del 25% al 49,99% que en aquellos en los que dichos niveles eran del 50% al 74,99% y un 28% (IC: 0,56; 0,92) menos prevalente en municipios con niveles de cobertura del 25% al 49,99% en comparación con niveles superiores al 75% (Tabla 4).

La implementación de procedimientos de vigilancia activa y continua de pacientes sometidos a seguimiento fue un 24% (IC: 0,62; 0,94) menos prevalente en municipios con niveles de cobertura de la ESF del 25% al 49,99% que en aquellos en los que dichos niveles eran superiores al 75%. A su vez, revisar los síntomas y seguir la evolución del cuadro clínico cada 48 horas (preferentemente por teléfono) fue un 32% (IC: 0,54; 0,87) menor en municipios con niveles de cobertura de la ESF del 25% al 49,99% que en aquellos con cobertura superior al 75% (Tabla 4).

En paralelo, verificar la disponibilidad de camas en el hospital de referencia para derivar pacientes que precisaban ser internados antes de derivarlos alcanzó una prevalencia un 45% (IC: 0,38; 0,81) menor en municipios con niveles de cobertura del 25% al 49,99% que en aquellos en los que dichos niveles eran superiores al 75% (Tabla 4).

**Tabla 4-:** Comparaciones entre los municipios según cobertura de la ESF*, con respecto a las medidas para manejar casos leves, al monitoreo y la vigilancia activa y continua, y a verificar la disponibilidad de camas antes de derivar pacientes. São Carlos, SP, Brasil, 2022

**Variable**	**Comparaciones**					
	**25%-49,99% vs 50%-74,99%**		**25%-49,99% vs >75%**		**50%-74,99% vs >75%**	
	**RP** ^†^ **(IC95%)** ^‡^	**Valor p** ^§^	**RP** ^†^ **(IC95%)** ^‡^	**Valor p** ^§^	**RP** ^†^ **(IC95%)** ^‡^	**Valor p** ^§^
¿Qué medidas se adoptaron para manejar los casos leves de COVID-19? ^||^						
Pauta de reposo	0,98 (0,92; 1,04)	0,44	0,99 (0,94; 1,04)	0,68	1,01 (0,97; 1,05)	0,54
Pauta de hidratación	0,97 (0,93; 1,02)	0,25	0,98 (0,94; 1,02)	0,32	1,01 (0,97; 1,04)	0,72
Pauta de alimentación	0,93 (0,87; 0,99)	0,03	0,95 (0,9; 1,01)	0,08	1,02 (0,97; 1,08)	0,41
Aislamiento domiciliario	0,97 (0,92; 1,01)	0,16	0,97 (0,92; 1,01)	0,17	1 (0,98; 1,03)	0,93
Analgésicos	0,88 (0,76; 1,01)	0,06	0,94 (0,84; 1,06)	0,32	1,08 (0,98; 1,18)	0,12
Antipiréticos	0,89 (0,76; 1,03)	0,12	0,92 (0,81; 1,05)	0,22	1,04 (0,94; 1,15)	0,41
Aislamiento domiciliario por 14 días a partir de la fecha en la que se iniciaron los síntomas	1,07 (0,91; 1,26)	0,40	1,09 (0,99; 1,2)	0,08	1,02 (0,88; 1,17)	0,81
¿La unidad de salud ofreció Teleasistencia para monitorear casos leves de COVID-19?						
No	-	-	-	-	-	-
Sí	0,73 (0,55; 0,98)	0,04	0,72 (0,56; 0,92)	<0,01	0,98 (0,82; 1,18)	0,85
¿La unidad de salud implementa procedimientos de vigilancia activa y continua en el caso de pacientes sometidos a seguimiento?						
No	-	-	-	-	-	-
Sí	0,83 (0,65; 1,07)	0,15	0,76 (0,62; 0,94)	<0,01	0,91 (0,78; 1,07)	0,26
¿La unidad de salud revisa los síntomas y sigue la evolución del cuadro clínico cada 48 horas (preferentemente por teléfono), solicitando consultas presenciales cuando es necesario realizar un examen físico?						
No	-	-	-	-	-	-
Sí	0,82 (0,58; 1,16)	0,27	0,68 (0,54; 0,87)	<0,01	0,83 (0,63; 1,08)	0,17
¿La unidad de salud verifica si el hospital de referencia para casos de internación por COVID-19 tiene suficientes camas disponibles para recibir a los pacientes que precisan ser hospitalizados antes de derivarlos?						
No	-	-	-	-	-	-
Sí	0,68 (0,44; 1,03)	0,07	0,55 (0,38; 0,81)	<0,01	0,82 (0,67; 1)	0,05

*ESF = Estrategia de Salud de la Familia; ^†^RP = Razón de Prevalencia; ^‡^IC95% = Intervalo de Confianza del 95%; ^§^Valor p = Nivel de significancia; ^||^Es posible que una misma persona haya dado más de una respuesta

## Discusión

Los resultados de este estudio demuestran que la cobertura de la ESF influenció la reorganización del proceso de trabajo durante la fase crítica de la pandemia del COVID-19. Los municipios en los que dicha cobertura era inferior al 25% presentaron resultados de mayor prevalencia en la implementación de medidas para prevenir el contagio del COVID-19, además de la reorganización del manejo clínico de casos leves y del proceso de trabajo.

En este contexto, los municipios más poblados tienden a tener menor cobertura de la ESF que los más pequeños. Esta cobertura reducida en grandes centros se debe a la mayor complejidad y densidad poblacional, lo que dificulta implementar y universalizar la APS. No obstante, esto no indica que no haya iniciativas, ya que muchas municipalidades compensan este déficit con mayor variedad de servicios y redes más complejas y robustas^(20)^.

Por otro lado, los municipios pequeños tienden a tener mayor cobertura de la ESF, al igual que en el caso de áreas vulnerables de municipios de mediano y gran porte^(20)^, que fueron los más afectados por el avance de la enfermedad en la fase crítica de la pandemia. Durante este período fue necesario que la administración pública implementase acciones más rápidas y efectivas para reorganizar flujos de APS y derivar casos con mayor complejidad clínica debido al COVID-19.

Los resultados de este estudio sugieren que los municipios de mayor porte y con menor cobertura de la ESF reorganizaron los servicios de APS de forma más práctica para optimizar los recursos disponibles, generalmente con baja densidad tecnológica y escasez de personal. En este escenario, los municipios paulistas con más de 50.000 habitantes dispusieron que todos los servicios de APS atendieran pacientes con COVID-19 disponiendo o no de unidades exclusivas para combatir la enfermedad^(21)^, imponiendo así a estos profesionales la inherente necesidad de adaptarse a la realidad de aquel momento. De forma similar, diversas ciudades de América Latina (especialmente en Chile y Colombia) reorganizaron la asistencia provista a pacientes con COVID-19 en unidades exclusivas, aunque también aprovechando la estructura ya existente de la APS^(22)^.

Entre las medidas adoptadas para reorganizar las unidades de la ESF se destacan acciones de prevención, reasignación de alas para pacientes sintomáticos y reorganización del flujo interno; todas estas medidas contribuyeron a controlar el COVID-19. En la fase inicial, medidas como el aislamiento, la cuarentena, el distanciamiento físico y el control de la circulación fueron eficaces para reducir la contaminación, ayudando a achatar la curva de transmisión^(23)^. Países como Italia, Francia, Alemania y España (que adoptaron un *lockdown* riguroso y extensas campañas de pruebas de detección) redujeron la letalidad y controlaron las mutaciones virales. En oposición, nuevas mutaciones del virus^(24)^ surgieron en Suecia, donde el *lockdown* fue más flexible.

Como el COVID-19 se transmite por las vías respiratorias (especialmente en lugares muy concurridos), fue esencial reorganizar el flujo de los servicios de salud. Separar pacientes con sospecha o confirmación de la enfermedad de los demás redujo el riesgo de transmisión. Esta reorganización también permitió un manejo más seguro, protegiendo así a profesionales y pacientes por igual y garantizando la continuidad de los servicios de forma segura y eficiente^(25)^. En Canadá, la rápida instalación de centros de aislamiento en las unidades permitió lograr buenos resultados al inicio mismo de la pandemia^(26)^. En Francia se crearon áreas de espera y pasillos específicos para pacientes sintomáticos en los servicios de APS^(16)^.

Se destaca que los municipios con menor cobertura de la ESF, de mayor porte y con mayor densidad poblacional pueden haber centralizado la atención de personas con síntomas de la enfermedad y/o con COVID-19 en ciertos servicios, reorganizando las unidades de la ESF para garantizar la continuidad de la asistencia y preservar la integralidad, la longitudinalidad y el acceso durante la emergencia sanitaria. Además, estos municipios tenían mayor capacidad para organizar los servicios en todos los niveles, con más posibilidades de movilizar equipamientos, suministros, personal y alternativas en las funciones de los servicios de salud^(27)^.

La emergencia del COVID-19 exigió que los sistemas de salud implementaran acciones rápidas a nivel global. Dichas acciones se dirigieron en un primer momento a garantizar la disponibilidad de camas para la población^(28)^; sin embargo, simultáneamente o poco después de la reorganización al nivel hospitalario, fue necesario restructurar inmediatamente los servicios de APS porque los hospitales se superpoblaron, como el colapso que se observó inicialmente en Italia^(29)^; también se verificó que la mayoría de los casos carecían de complejidad o que sería posible capilarizar la asistencia a partir de los servicios de PAS, que habían permanecido subutilizados hasta ese momento^(30)^.

Vale destacar que la APS debe ofrecer gran capacidad de resolución en materia de las necesidades básicas de salud y ser eficaz para reducir la cantidad de internaciones por patologías sensibles. Sin embargo, durante la pandemia, su subutilización agravó la crisis ya existente en un sistema históricamente subfinanciado, contribuyendo así a la escasez de recursos. La fragilidad de esta base puso en riesgo el monitoreo de los casos de COVID-19 y la implementación de acciones efectivas de búsqueda activa y vigilancia, lo que aumentó la cantidad de internaciones y sobrecargó a los servicios de emergencia. Haber fortalecido la APS podría haber generado una respuesta más eficiente y mitigado el efecto de la pandemia en Brasil^(28)^.

Los atributos de la APS (incluida la ESF), como su conexión con el territorio, su vínculo con los usuarios y la longitudinalidad de la atención provista, se exploraron escasamente en muchos municipios de Brasil, principalmente durante el primer año de la pandemia. Durante la pandemia se verificó una significativa reducción en el uso de servicios de salud preventivos y terapéuticos, atribuida a las restricciones en materia de movilidad, al distanciamiento físico y al miedo a infectarse^(31)^.

A pesar del atraso en redireccionar la APS para hacer frente a la pandemia, los servicios de fueron adaptando y fortaleciendo gradualmente hasta asumir el rol central en la asistencia durante la emergencia sanitaria. La capacidad de adaptación e innovación que demostró la APS permitió reorganizar eficientemente el flujo de pacientes y gerenciar protocolos clínicos nuevos, reforzando así la importancia de cualificar a la APS para garantizar la continuidad de la atención y la gestión de patologías ajenas a la emergencia^(32)^.

Durante la fase crítica de la pandemia, la adaptación de la APS y la intensificación de la carga de trabajo expusieron diferencias significativas entre los municipios debido a la ausencia de directrices nacionales. Diversas desigualdades locales y/o regionales en la estructura de la APS (como recursos humanos, acceso a insumos, tecnologías y capacidad de coordinación) acentuaron las respuestas desiguales ante la crisis. Los municipios con mayor cobertura de la ESF, gestión estructurada e integración con los servicios de vigilancia epidemiológica demostraron mejores respuestas, mientras que localidades con baja densidad técnica o debilidades históricas afrontaron más dificultades al momento de organizar la asistencia y el manejo clínico del COVID-19. Estas diferencias se agravaron debido a la falta de protocolos unificados, lo que obligó a gerentes y profesionales a tomar decisiones locales en medio de incertezas^(3,33)^.

Además de la gestión clínica, el manejo del dolor y las pautas adecuadas sobre alimentación e ingesta líquida no pueden disociarse de la educación para la salud, uno de los pilares fundamentales de la ESF. Durante la pandemia, la educación para la salud fue esencial no solo para promover acciones de prevención (como el uso de mascarillas o el distanciamiento físico) sino también para combatir la desinformación sobre el virus y las vacunas, brindar pautas orientativas sobre identificación de síntomas y medidas preventivas en el hogar, reducción del estigma asociado a la infección y propuestas de apoyo para la salud mental^(34)^.

Cabe destacar que la ESF ofrece asistencia dirigida a grupos prioritarios definidos por políticas públicas de salud, a saber: niños, ancianos, mujeres y personas con hipertensión, diabetes, obesidad y problemas de salud mental^(35)^. En este contexto, se considera que el COVID-19 es una enfermedad emergente y endémica actualmente y que se lo debe incluir en el arsenal de actividades de la ESF; es decir, los gerentes deben buscar medios para capacitar a los profesionales de la salud frente a una nueva demanda para que puedan ofrecer asistencia integral y resolutiva (además de las acciones de vigilancia), independientemente de la cobertura poblacional de cada municipio.

Los resultados de este estudio también demostraron que la vigilancia de casos posibles o confirmados fue más frecuente en localidades con mayor cobertura de la ESF. Uno de los motivos de este logro puede estar relacionado con la composición de las unidades de la ESF, especialmente el desempeño de los Agentes Comunitarios de Salud (ACS). El trabajo de los ACS durante la pandemia fue determinante para el éxito de la vigilancia comunitaria, ya que estos profesionales promovieron asistencia territorializada y continua, incluso ante situaciones adversas y de alta demanda, expandiendo el alcance de las acciones de vigilancia, especialmente en áreas vulnerables con acceso restringido a los servicios de salud^(36-37)^.

La eficacia del trabajo realizado por los equipos de la ESF en la vigilancia activa de casos de COVID-19 (e incluso la propia asistencia dirigida a estos pacientes) estuvo asociada a la utilización de tecnologías de la información y la comunicación como la Telemedicina y diversas plataformas digitales, lo que facilitó implementar consultas virtuales y monitoreo remoto y diseminar información, aumentando así la capacidad para acceder a la atención y su eficiencia. Estas acciones integradas fueron fundamentales para controlar la propagación del virus y garantizar la continuidad de la atención^(38)^. En su mayoría, los municipios con mayores niveles de cobertura de la ESF son de mediano y pequeño porte, pueden tener un solo servicio de salud en muchos casos y ostentan mejor control y eficacia en cuanto a acciones de vigilancia, debido a la posibilidad de acceder a muchos domicilios, a su vínculo con la población y a la demanda de servicios. En consecuencia, esta característica puede justificar la mayor adhesión a las medidas de vigilancia, al monitoreo de la evolución del cuadro clínico de cada paciente y al uso de la Telemedicina y el Telemonitoreo en estos servicios.

Promovidas con mayor prevalencia en los municipios con gran cobertura de la ESF, las acciones de vigilancia sanitaria efectivas no solo favorecieron el monitoreo y control del COVID-19 sino que también contribuyeron a derivar correctamente los casos graves, optimizando así la utilización de las camas de hospital disponibles. En este contexto se destaca la importancia de la articulación entre los diferentes puntos de la Red de Atención de la Salud (RAS), con énfasis en la integración entre la APS y los servicios de mediana y alta complejidad puesto que, combinado a la capacitación y cualificación de los profesionales de la salud, el flujo continuo de información demostró ser esencial para aprovechar eficazmente los recursos existentes, sobre todo en situaciones de escasez^(39)^. Además, en otro escenario (Noruega) se verificó cierta integración entre servicios de APS y otros servicios de salud por medio de protocolos y directrices unificados, a fin de garantizar atención segura para todos los pacientes^(40)^.

No obstante, la pandemia agravó las desigualdades ya existentes en la distribución de camas de hospital entre los municipios de Brasil, ya que regiones con menores condiciones socioeconómicas sufrieron mayores dificultades para acceder a internaciones, lo que derivó en coeficientes más altos de hospitalizaciones tardías y mortalidad. La centralización de los recursos hospitalarios en las capitales y en ciudades de mediano porte dificultó el acceso oportuno a servicios de cuidados intensivos en el caso de poblaciones en áreas remotas o periféricas. Esta situación expuso fragilidades estructurales e históricas del Sistema Único de Salud (SUS) y puso de manifiesto la urgencia de redistribuir los recursos de salud más equitativamente, en especial en lo que se refiere a la infraestructura hospitalaria^(41)^.

Dado este escenario, se vuelve imprescindible fortalecer la APS, no solo como ingreso al sistema sino también como coordinadora de la atención, con mayor capacidad resolutiva y estrategias de manejo clínico eficaces. Combinado con protocolos clínicos bien definidos, el desempeño cualificado de los equipos de la ESF puede contribuir a un cribado preciso de los casos y a garantizar que las derivaciones al nivel hospitalario se realicen de forma oportuna, racional y en función de la gravedad de cada caso, contribuyendo así a reducir la sobrecarga de los hospitales y los desenlaces adversos^(42-43)^.

Finalmente, los resultados de este estudio demuestran que la presencia de equipos de la ESF permitió implementar una respuesta ágil y adaptable, facilitando así el manejo de pacientes con COVID-19, la promoción de medidas preventivas y el uso de tecnologías de la comunicación como la Telemedicina. Se destaca que estas tecnologías siguieron siendo significativas después de la pandemia, especialmente en lugares con mayor madurez digital, formación digital en salud e infraestructuras favorables, lo que representa un gran legado, no solo en Brasil sino también en el mundo^(44)^.

Al igual que en Brasil, la pandemia también aceleró transformaciones en la APS de Canadá, impulsando la expansión de iniciativas digitales como programación de consultas en línea, servicios de emergencias y clínicas especializadas de COVID-19, además de la Teleasistencia. Estos cambios dejaron un legado en lo que se refiere a la consolidación de la Telesalud y a la necesidad de implementar modelos más ágiles e integrados que puedan responder ante las crisis sin poner en riesgo el acceso a la atención de la salud^(45)^. Ambos casos refuerzan el rol estratégico de la APS y de la infraestructura digital en situaciones de emergencia sanitaria.

Este estudio presenta limitaciones inherentes a su diseño transversal, como la ausencia de causalidad y el sesgo de prevalencia. El predominio de municipios de la región Sudeste en la muestra puede haber introducido cierto sesgo de prevalencia. Además, es posible que haber utilizado formularios de auto-respuesta para recolectar los datos haya generado un sesgo de respuesta debido a posibles interpretaciones erróneas de las preguntas y a la sobre o subestimación de la información provista por los participantes. No obstante, la muestra incluye representantes de todos los estados y ofrece resultados uniformes y robustos sobre cómo se reorganizó la APS en Brasil durante la fase crítica de la pandemia, en relación con la cobertura de las unidades de la ESF.

## Conclusión

La ESF desempeñó un rol esencial durante la fase crítica de la pandemia del COVID-19 porque garantizó asistencia continua y eficaz a la población. El desarrollo de acciones preventivas, el monitoreo de casos leves y la orientación comunitaria contribuyeron a reducir la propagación del virus y a gestionar las necesidades de salud de la población. Además, la ESF ayudó a mantener la continuidad de la atención en el caso de patologías crónicas y otros requerimientos de salud, demostrando así su importancia en poder responder de forma adaptable y sustentable ante emergencias sanitarias.

Se sugiere desarrollar futuros trabajos de investigación en los que se evalúe el efecto de la reorganización de la APS durante la pandemia en el proceso de trabajo de los equipos a largo plazo. Además, los resultados ponen de manifiesto la importancia de diseñar y estandarizar protocolos que orienten la reorganización de los servicios de salud en situaciones de emergencia en salud pública.
